# Supplementary material for: Design, Synthesis and Structure—Activity Relationships of Phenylalanine-Containing Peptidomimetics as Novel HIV-1 Capsid Binders Based on Ugi Four-Component Reaction
Source: Molecules. 2022 Sep 14;27(18):5995. doi: 10.3390/molecules27185995 (PMC9502897; doi:10.3390/molecules27185995)
Supplement: Supplementary file 1 [file molecules-27-05995-s001.zip › supplementary information.pdf]

Supporting Information for

Original article

Design, Synthesis and Structure-Activity Relationships of Phenylalanine-Containing Peptidomimetics as Novel HIV-1 Capsid Binders Based on Ugi Four-Component Reaction

Xiangkai Ji[a], Jing Li[a], Prem Prakash Sharma[b], Xiangyi Jiang[a], Brijesh Rathi[b], Zhen Gao[a], Lide Hu[a], Dongwei Kang[a, c], Erik De Clercq[d], Simon Cocklin[e], Chuanfeng Liu[a], Christophe Pannecouque[d,*], Alexej Dick[e,*], Xinyong Liu[a, c,*] and Peng Zhan[a, c,*]

[a]*Department of Medicinal Chemistry, Key Laboratory of Chemical Biology (Ministry of Education), School of Pharmaceutical Sciences, Cheeloo College of Medicine, Shandong University, 44 West Culture Road, 250012 Jinan, Shandong, PR China*

[b]*Laboratory for Translational Chemistry and Drug Discovery, Department of Chemistry, Hansraj College, University of Delhi, Delhi 110007, India*

[c]*China-Belgium Collaborative Research Center for Innovative Antiviral Drugs of Shandong Province, 44 West Culture Road, 250012, Jinan, Shandong, PR China*

[d]*Rega Institute for Medical Research, Laboratory of Virology and Chemotherapy, K.U.Leuven, Herestraat 49 Postbus 1043 (09.A097), B-3000 Leuven, Belgium*

[e]*Department of Biochemistry & Molecular Biology, Drexel University College of Medicine, Philadelphia, PA, USA*

Cotent

1. **Molecular dynamics (MD) simulation** 2

2. **Surface plasmon resonance (SPR) assay** 3

8. **Original spectra of compounds** 4

MATERIAL AND METHODS OF MD

The coordinate structure of gag-polyprotein (PDB: 5TSX, 5HGL), was collected from RCSB website (https://www.rcsb.org) for ligand-protein complex interaction analysis. The computational work was performed using Schrodinger software.

Preparation of protein and ligand structure

These downloaded structures prepared prior to docking to remove structural errors [1]. The compounds I-19 and PF-74 were prepared by Ligprep tool prior to docking [2]. Schrödinger suite inbuilt Epik module was also used to predict the ionization states of all compounds at pH 7 ± 2 as well as tautomers generated [3]. This in-silico study was done under OPLS2005 forcefield.

Molecular docking of designed chemical library

Site specific molecular docking of both compounds against HIV-1 capsid protein performed at XP precision using the Glide module of Schrödinger suite [4]. The binding site of capsid protein (PDB: 5TSX) was defined in reference of PDB: 5HGL. The Van der Waals radii scaling factor and partial charge cutoff was 0.8 and 0.15 used for docking, respectively. The binding free energy for these three complexes were also calculated by prime mmGBSA [5].

Molecular Dynamics (MD) Simulation

In order to further verify the accuracy of docking observations, these both complexes of compound I-19 and PF-74 with capsid protein were selected for extensive 100ns MD simulation. Both the complexes were introduced into Desmond software to study the binding stability of both compounds within their respective complex [6]. These complexes were solvated in TIP3P water model and Na^+ ions are added to neutralize both complexes. *The stereo-chemical geometry of 5TSX protein residues was measured by Ramachandran map by procheck [7].*

References

1 Schrödinger Release 2020-1: Protein Preparation Wizard; Epik; Prime, S., LLC, New York, NY, 2016; Impact, Schrödinger, LLC, New York, NY, 2016; Prime, Schrödinger, LLC, New York, NY, 2020. (2020)

2 Schrödinger Release 2020-1: LigPrep, S., LLC, New York, NY, 2020. (2020)

3 Schrödinger Release 2020-1: Epik, S., LLC, New York, NY, 2020. (2020)

4 Schrödinger Release 2020-1: Glide, S., LLC, New York, NY, 2020. (2020)

5 Schrödinger Release 2020-1: Prime, S., LLC, New York, NY, 2020. (2020) Prime.

6 Schrödinger Release 2020-1: Desmond Molecular Dynamics System, D.E.S.R., New York, NY, 2020. Maestro-Desmond Interoperability Tools, Schrödinger, New York, NY, 2020. (2020)

7 Laskowski, R.A. et al. (1993) PROCHECK: a program to check the stereochemical quality of protein structures. 26 (2), 283-291

MATERIAL AND METHODS OF SPR

IgG b12 anti-HIV-1 gp120; was obtained through the NIH AIDS Reagent Program, Division of AIDS, NIAID, NIH: Anti-HIV-1 gp120 Monoclonal (IgG1 b12) from Dr. Dennis Burton and Carlos Barbas); p24 was produced in-house as previously described[1]. Briefly, a vector containing C-terminally His-tagged HIV-1_{NL4-3}CA (a gift from Dr. Eric Barklis, Oregon Health and Science University, Portland, OR) was transformed into BL21-Codon Plus (DE3)-RIL Competent Cells (Agilent Technologies, Wilmington, DE) and grown up in autoinduction ZYP-5052 medium overnight with shaking (225 rpm) at 30°C[2]. Bacterial cultures were spun down at 7000 rpm, and the supernatant was discarded. Cell pellets were resuspended in PBS and lysed via sonication. The

resultant supernatant was clarified and immediately applied to a Talon cobalt resin affinity column (Clonetech Laboratories, Mountain View, CA). Protein was eluted using 1X PBS with 250mM imidazole. Purified CA-H6 monomers were dialyzed overnight into 20mM Tris-HCl pH 8.0 at 4°C, concentrated to 120μM, flash-frozen in liquid nitrogen, aliquoted, and stored at -80°C. The CA hexamer was generated by introducing mutations at the following sites: A14C, E45C, W184A, and M185A through site-directed mutagenesis (Stratagene). While the A14C, E45C mutations stabilize the CA hexamer, the W184A, and M185A prevent further oligomerization of hexamers into CA cones and tubes. The CA hexamer construct was expressed and purified following the same protocol as described above. After purification, the CA-H6 hexamers were dialyzed into 200mM ß-ME followed by sequential dialyzes to remove the ß-ME to allow for hexamer assembly slowly.

[1] S. Kortagere, N. Madani, M.K. Mankowski, A. Schön, I. Zentner, G. Swaminathan, A. Princiotto, K. Anthony, A. Oza, L.J. Sierra, S.R. Passic, X. Wang, D.M. Jones, E. Stavale, F.C. Krebs, J. Martín-García, E. Freire, R.G. Ptak, J. Sodroski, S. Cocklin, A.B. Smith, 3rd, Inhibiting early-stage events in HIV-1 replication by small-molecule targeting of the HIV-1 capsid, J Virol, 86 (2012) 8472-8481.

[2] F.W. Studier, Protein production by auto-induction in high density shaking cultures, Protein Expr Purif, 41 (2005) 207-234.

3. Original spectra of compounds.

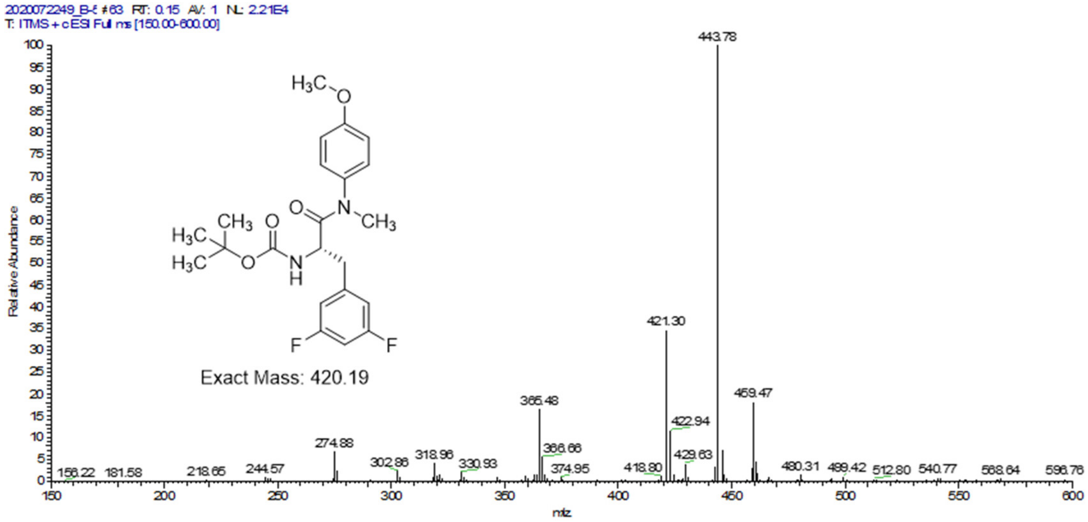

MS spectrum of **I-2**

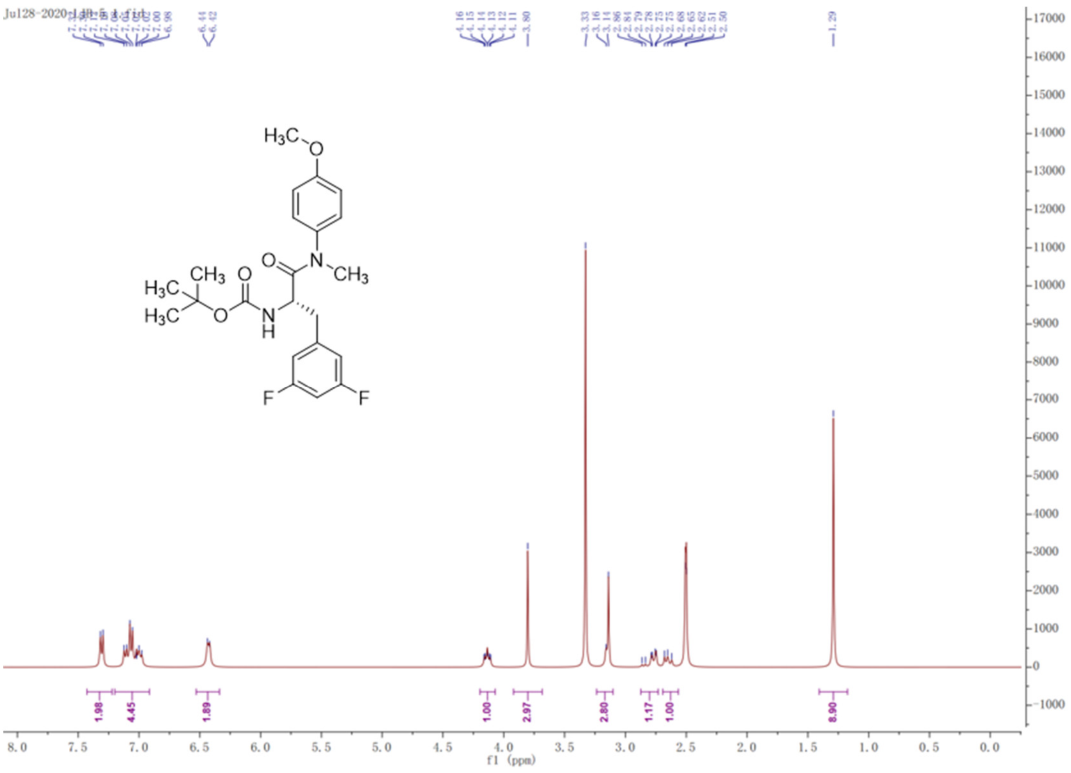

^{1}H-NMR of **I-2**

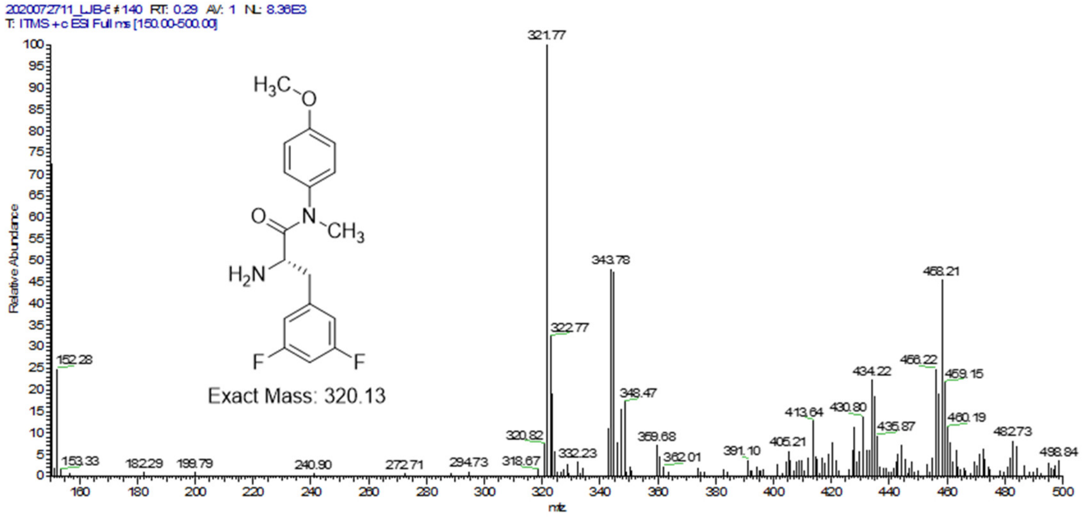

MS spectrum of **I-3**

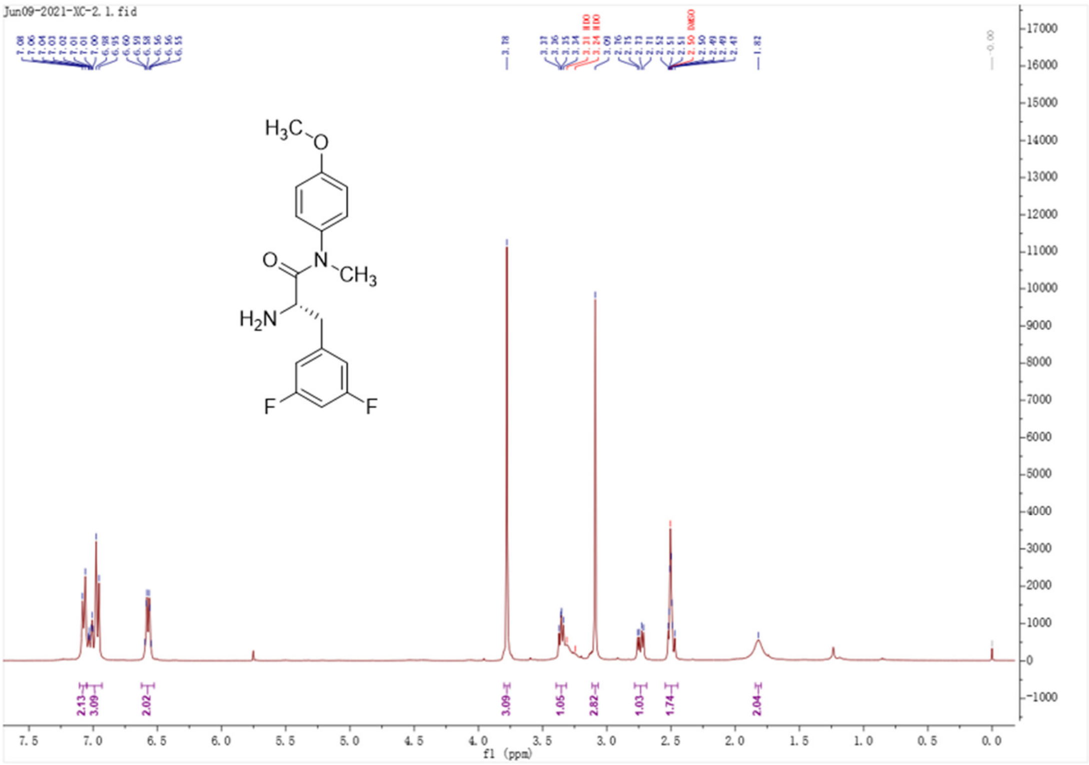

Jun09-2021-XC-2.1.fid
H3C
O
O
N
CH3
H2N
F
F
2.13
3.09
2.02
3.09
1.05
2.82
1.03
1.74
2.04
f1 (ppm)

^{1}H-NMR of **I-3**

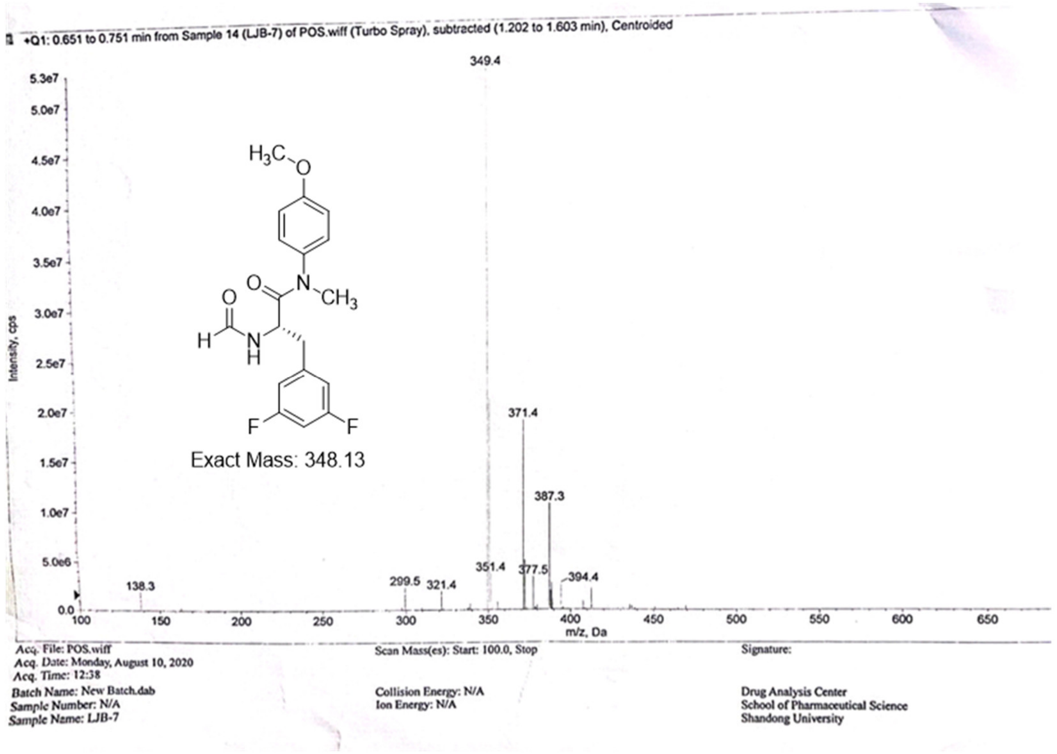

MS spectrum of **I-4**

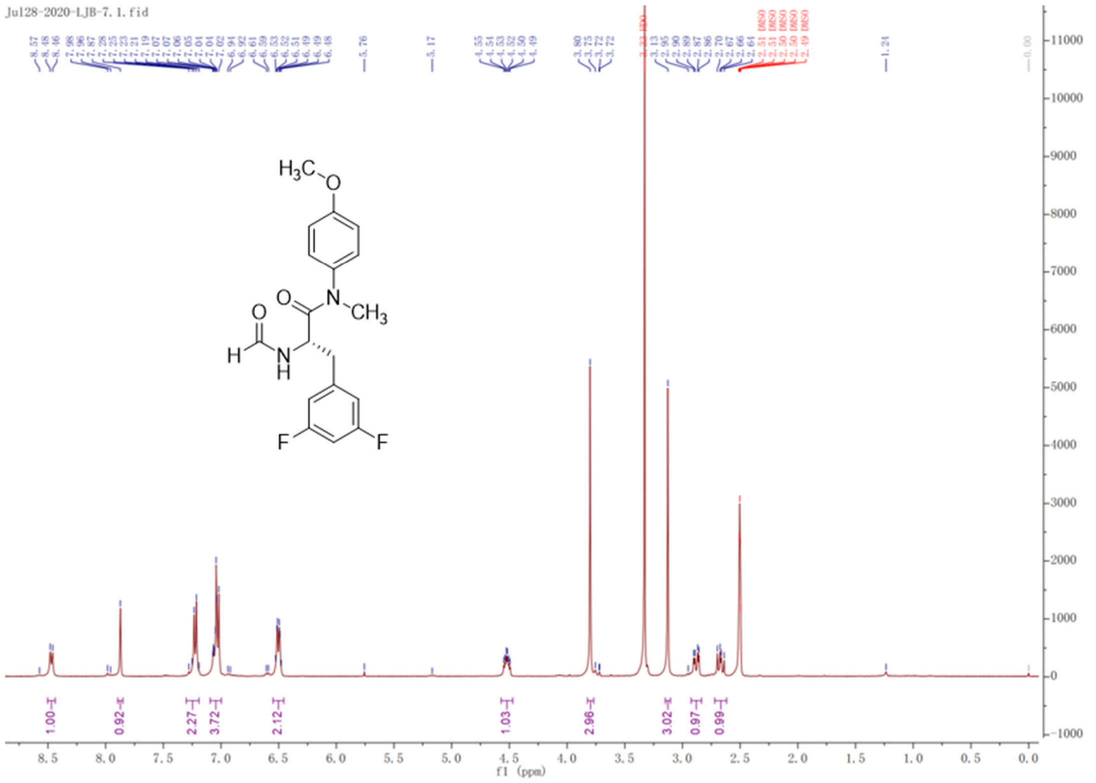

^{1}H-NMR of **I-4**

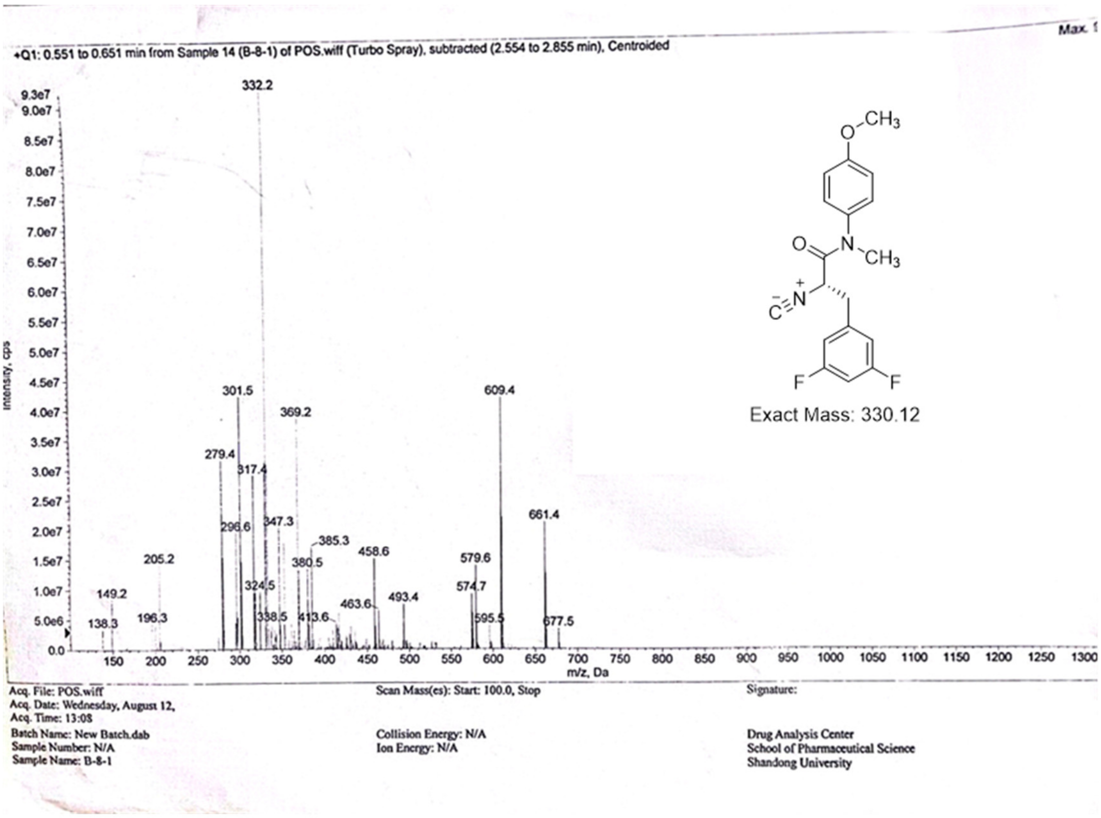

MS spectrum of **I-5**

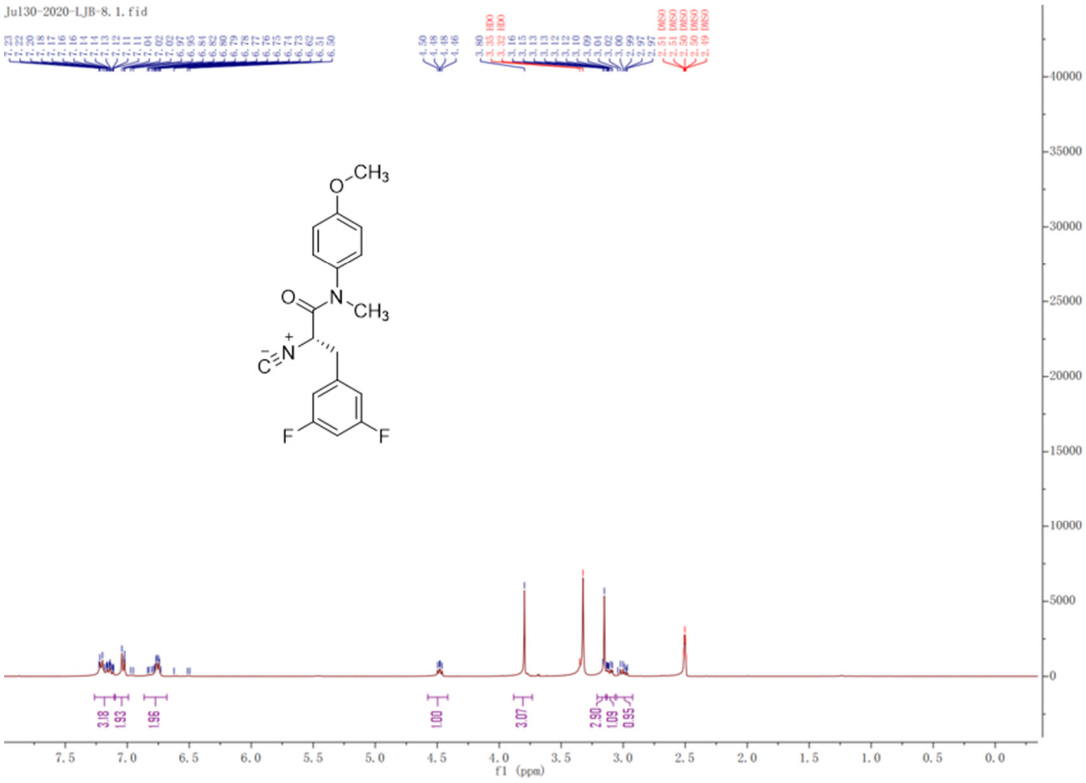

^{1}H-NMR of **I-5**

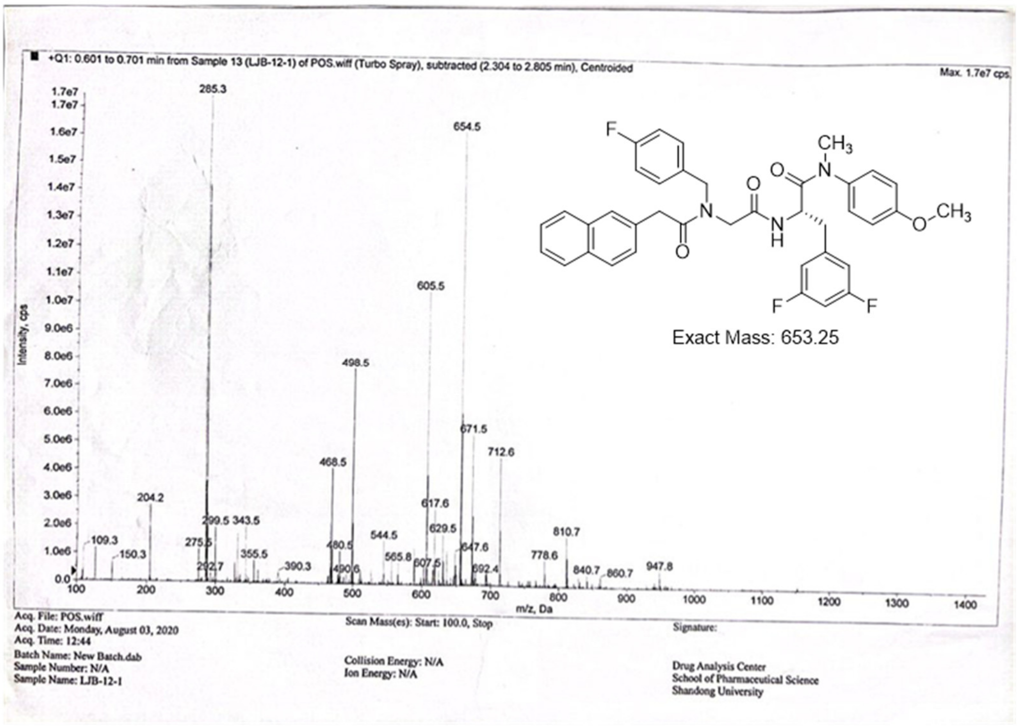

MS spectrum of **I-6**

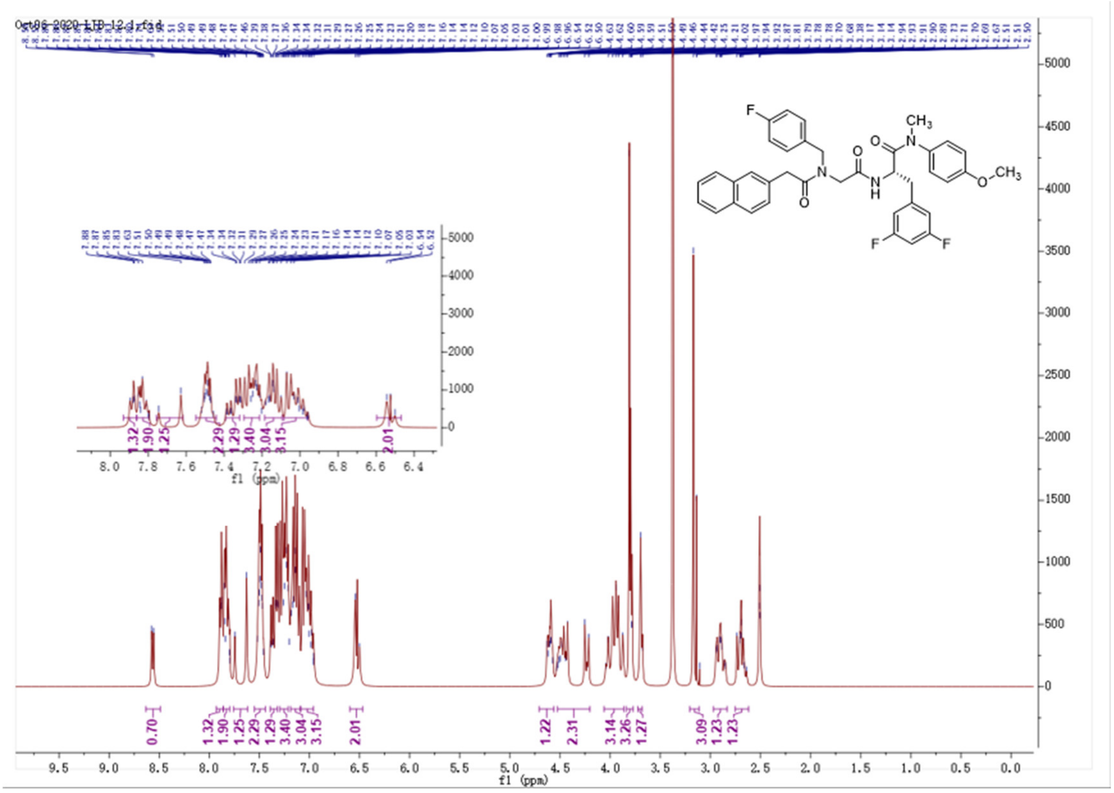

^{1}H-NMR of **I-6**

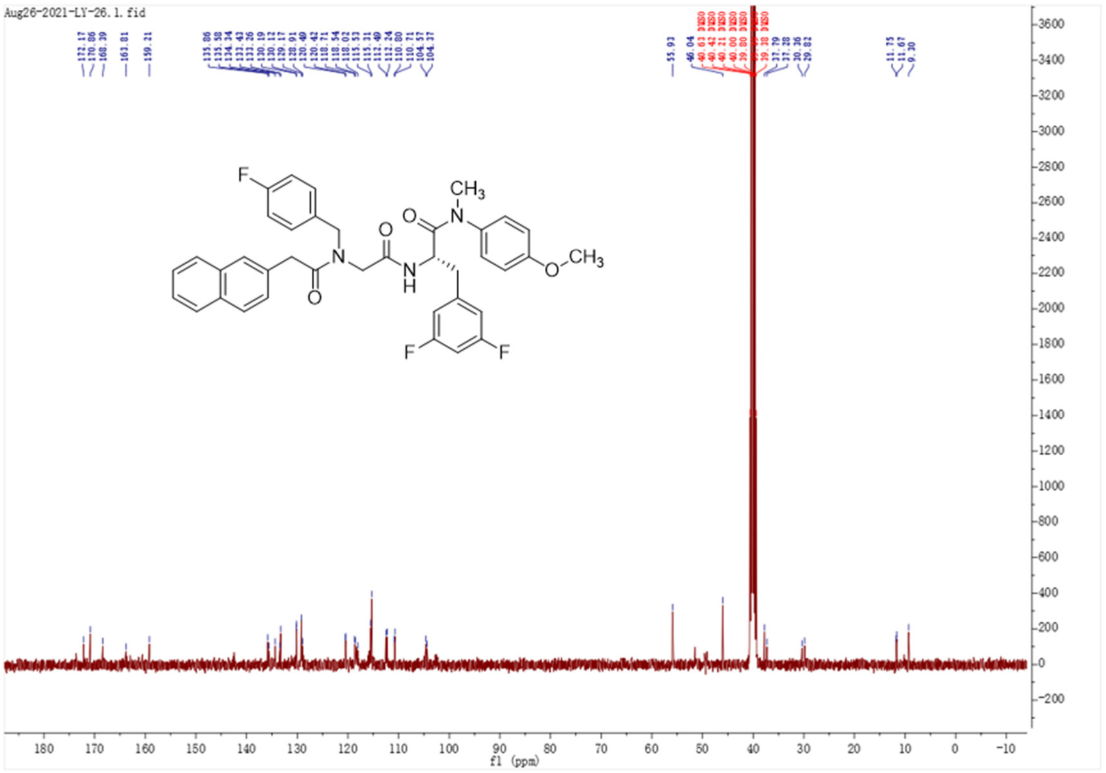

^{13}C-NMR of **I-6**

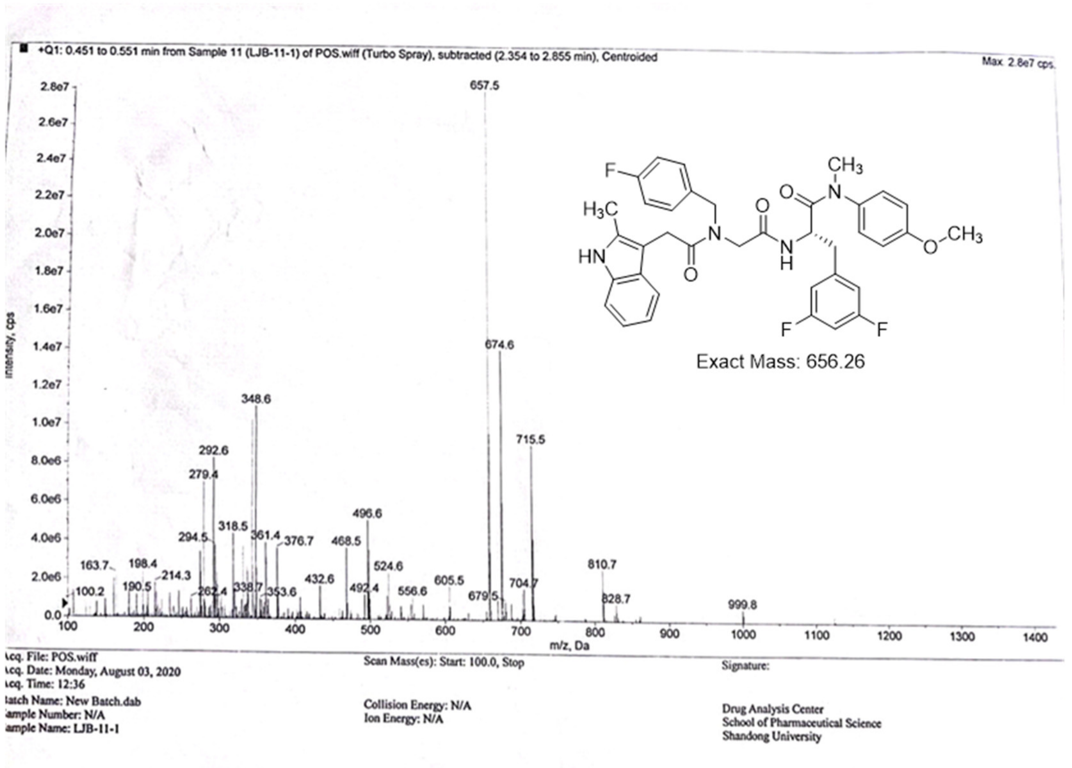

MS spectrum of **I-7**

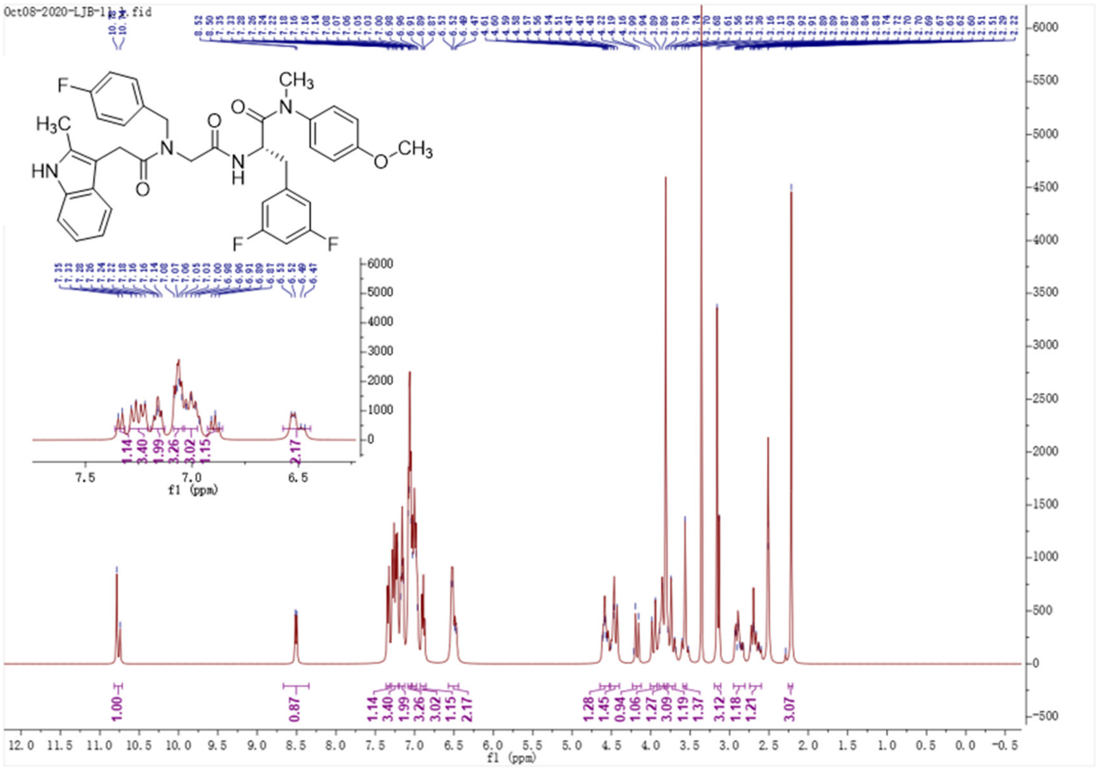

^{1}H-NMR of **I-7**

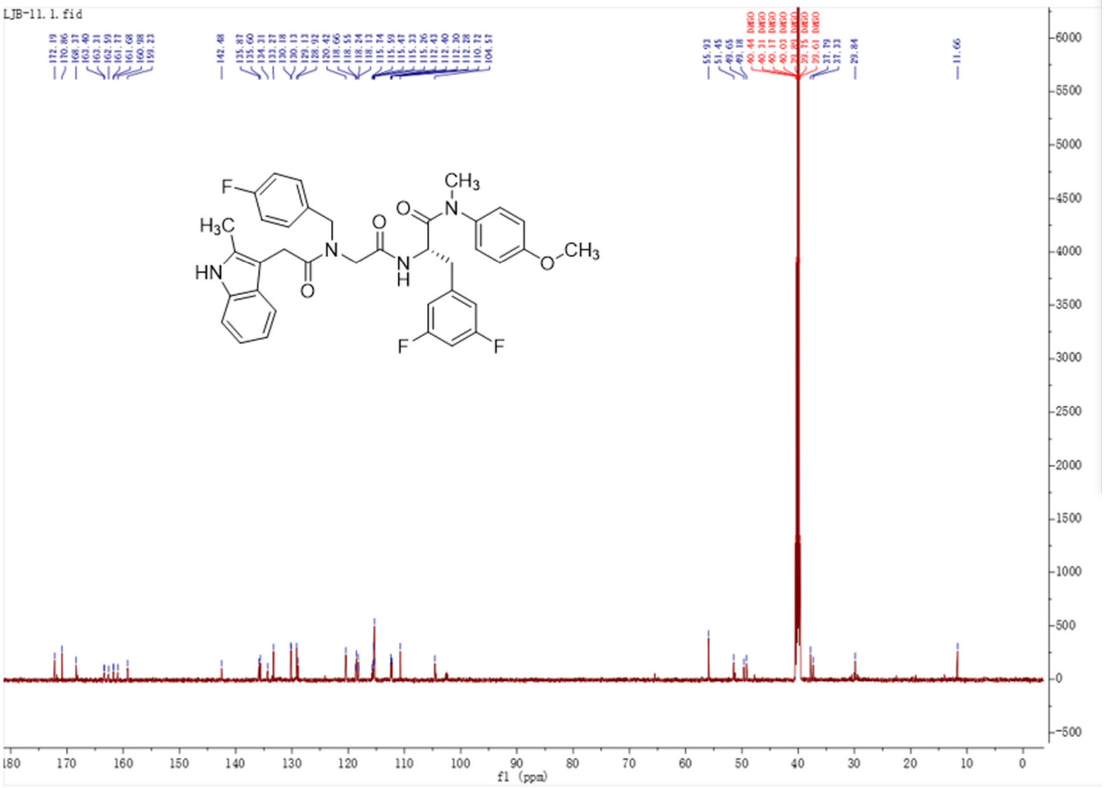

^{13}C-NMR of **I-7**

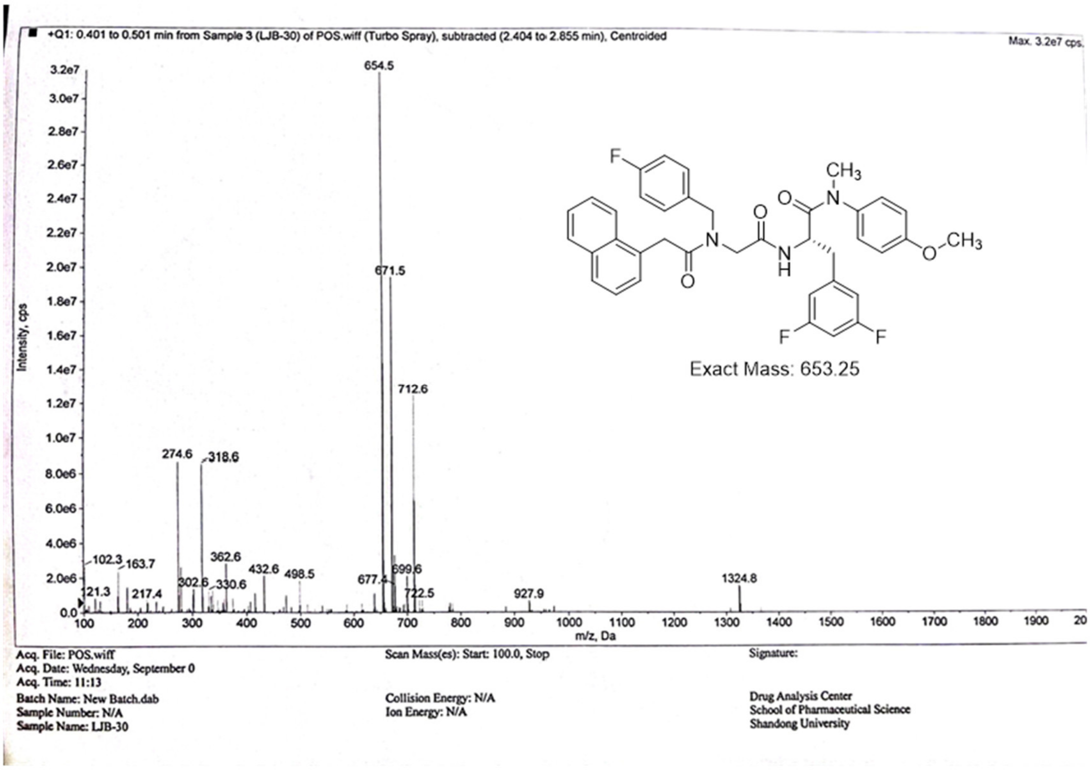

MS spectrum of **I-8**

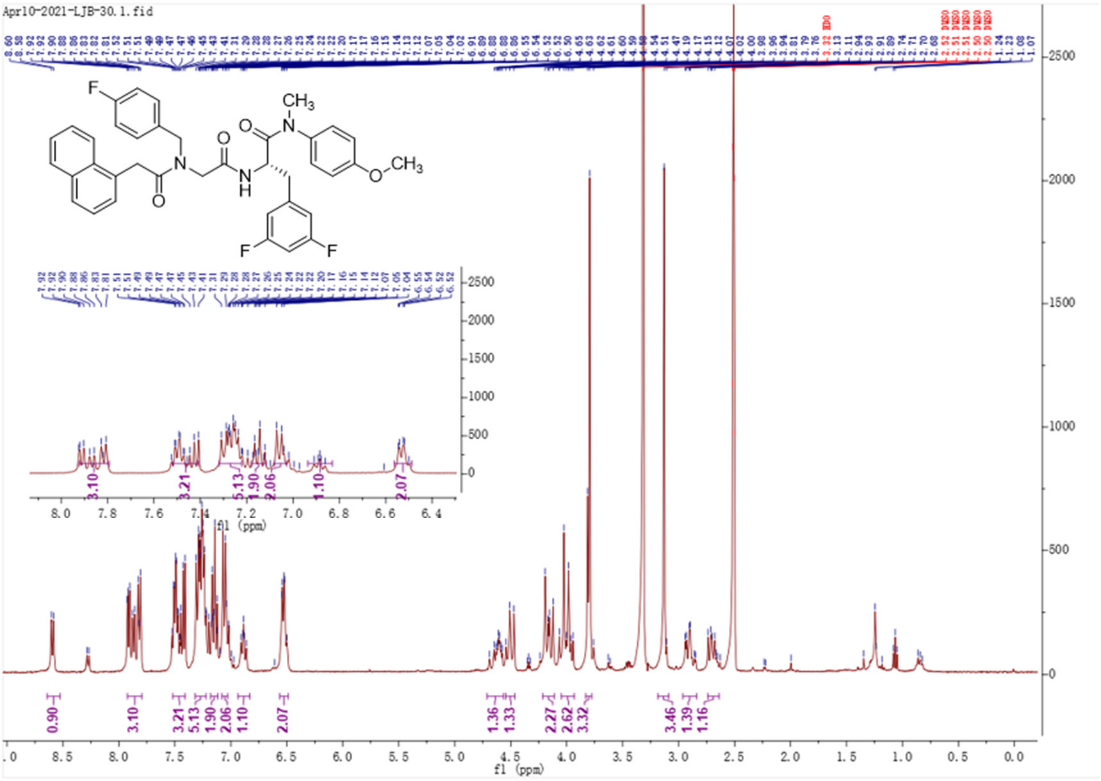

^{1}H-NMR of **I-8**

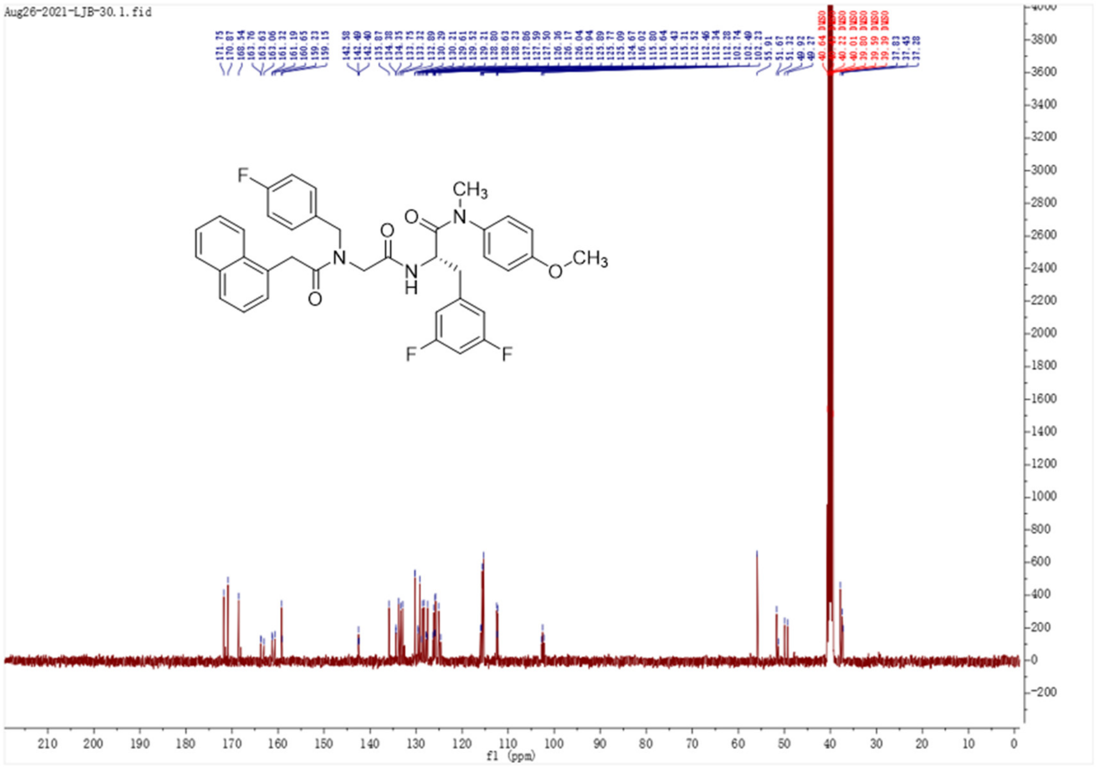

^{13}C-NMR of **I-8**

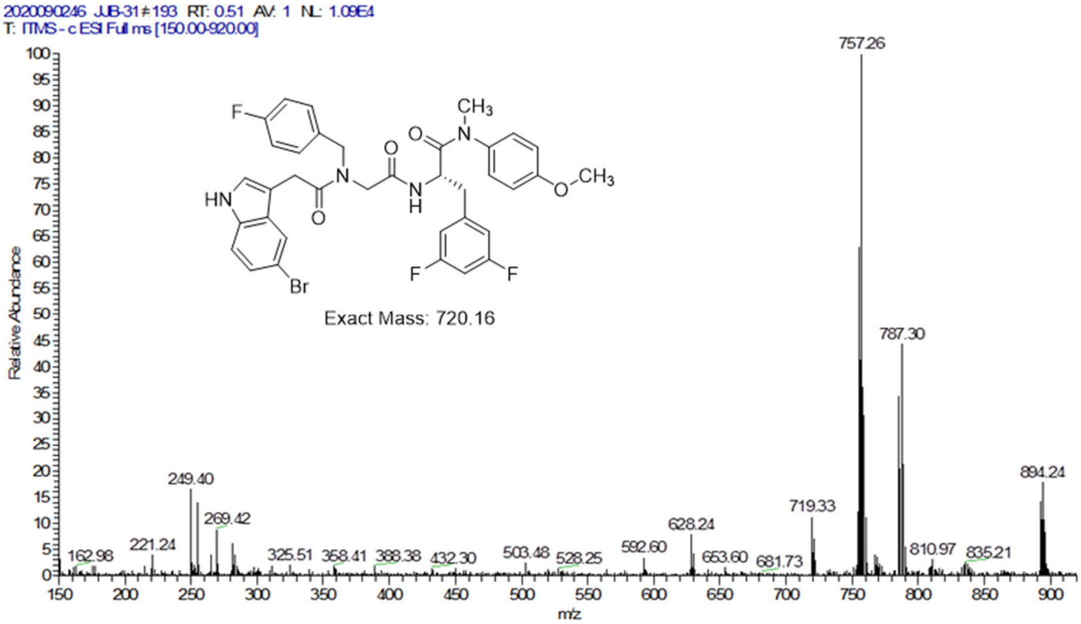

MS spectrum of **I-9**

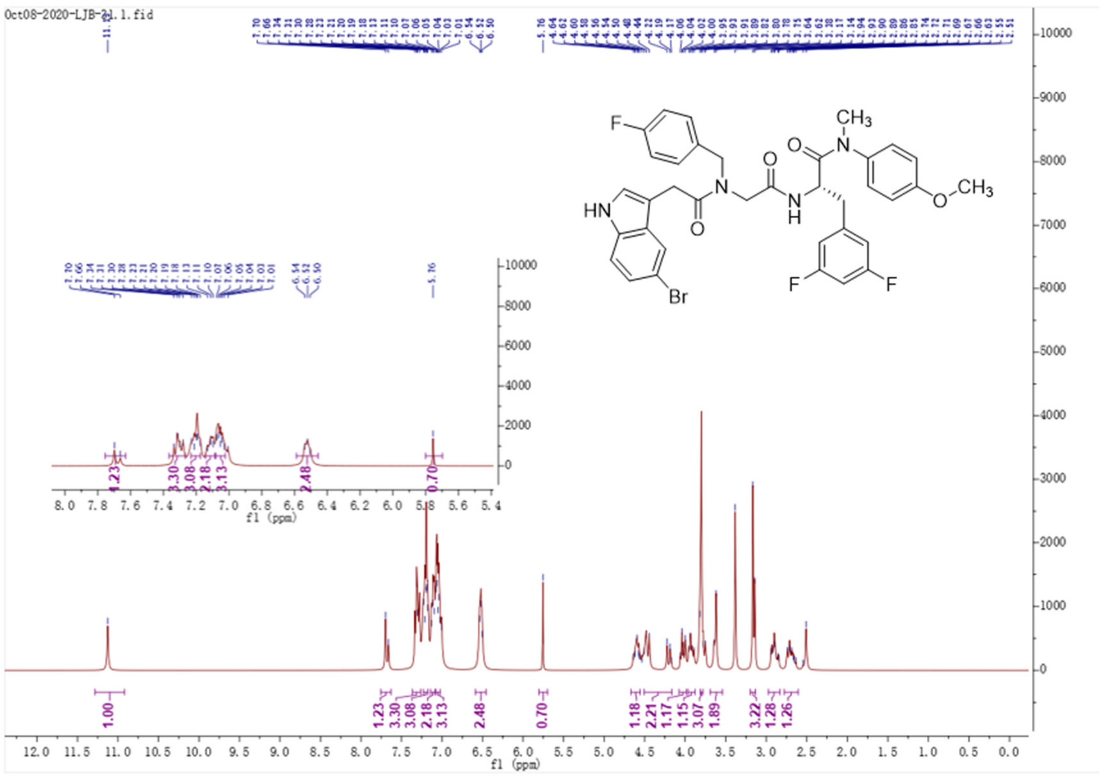

^{1}H-NMR of **I-9**

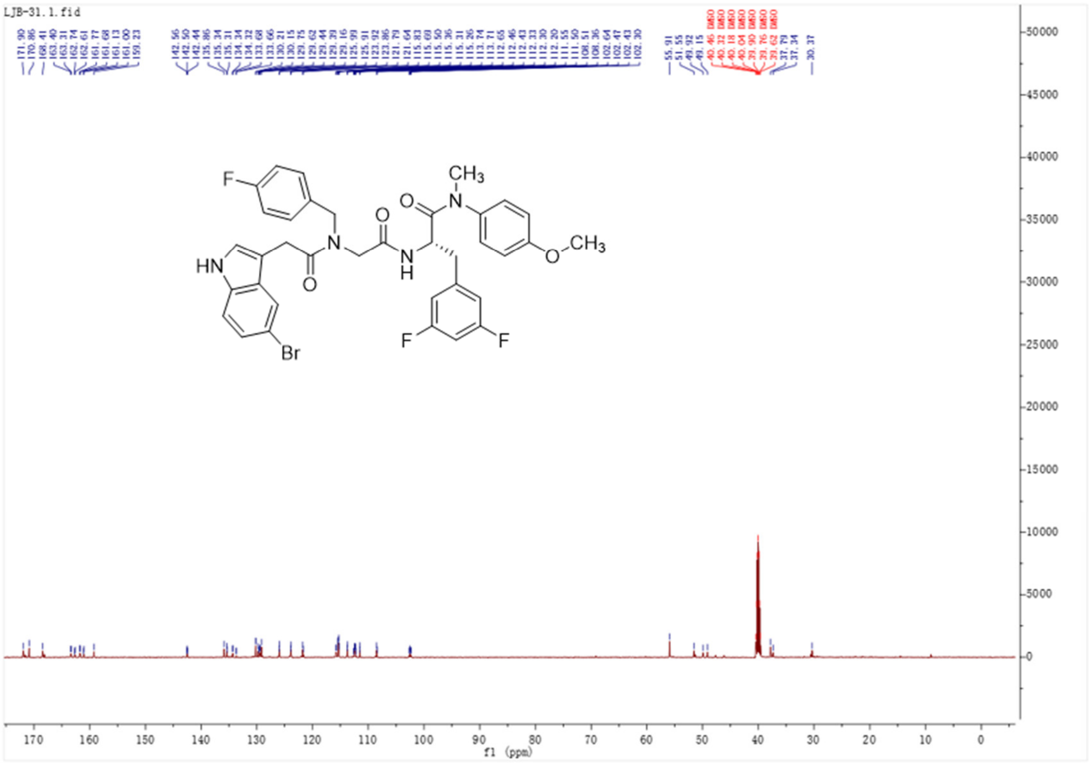

^{13}C-NMR of **I-9**

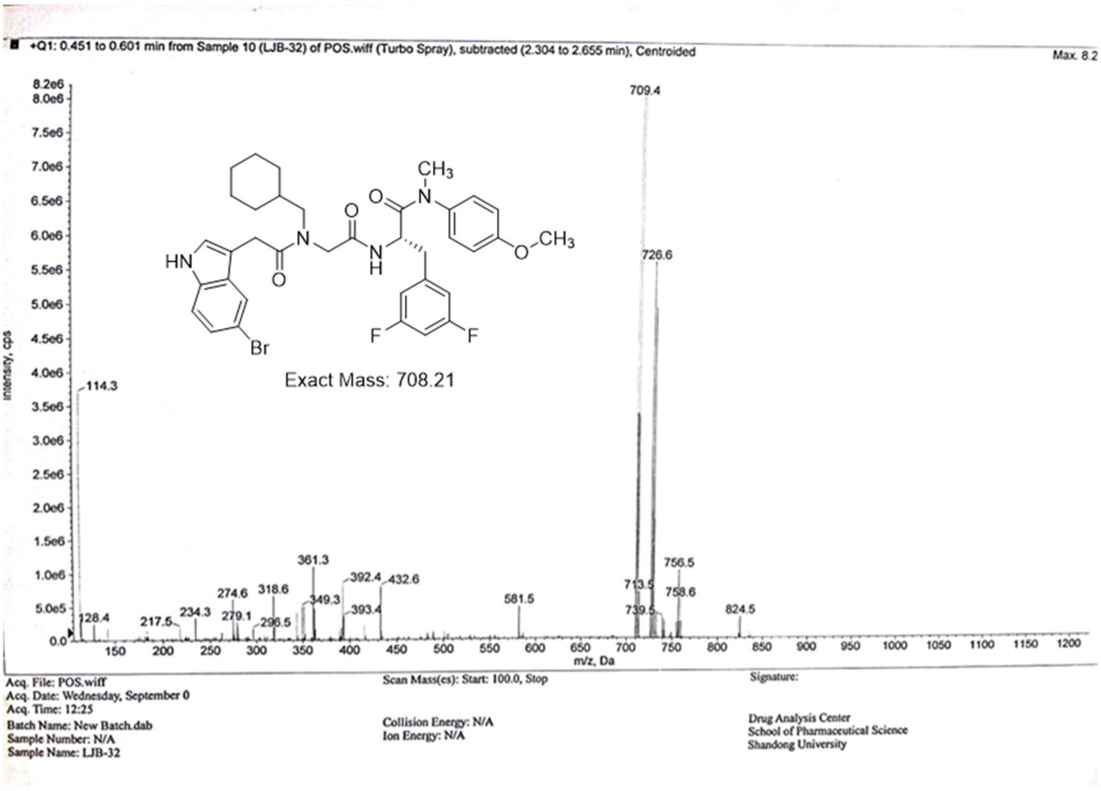

MS spectrum of **I-10**

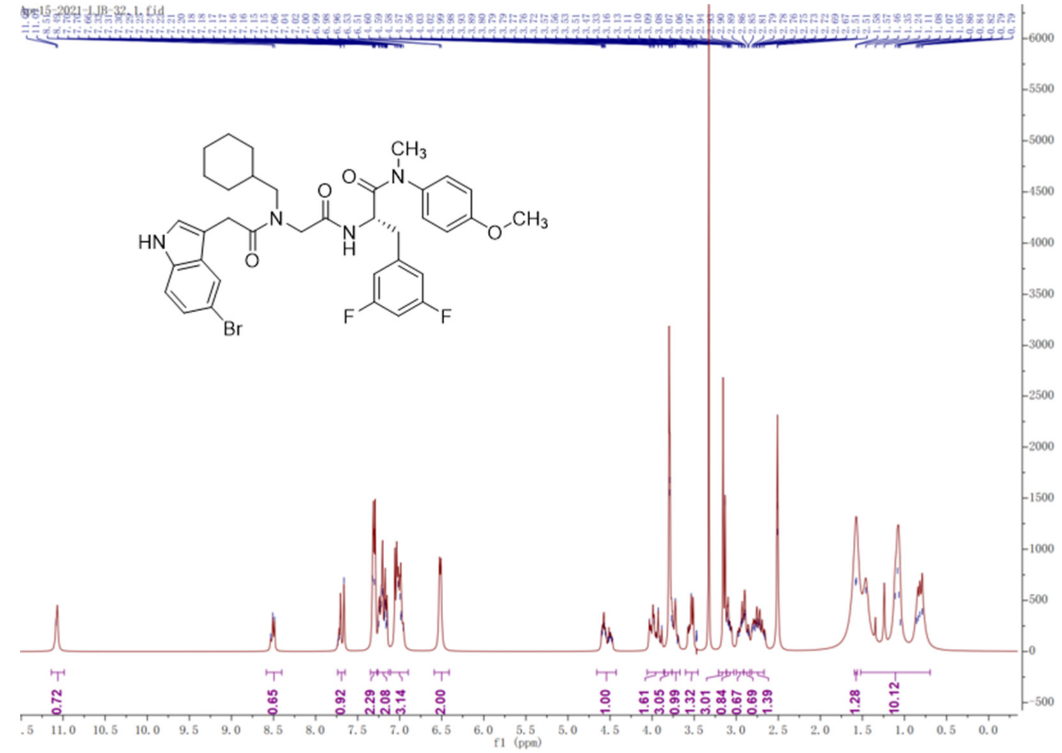

^{1}H-NMR of **I-10**

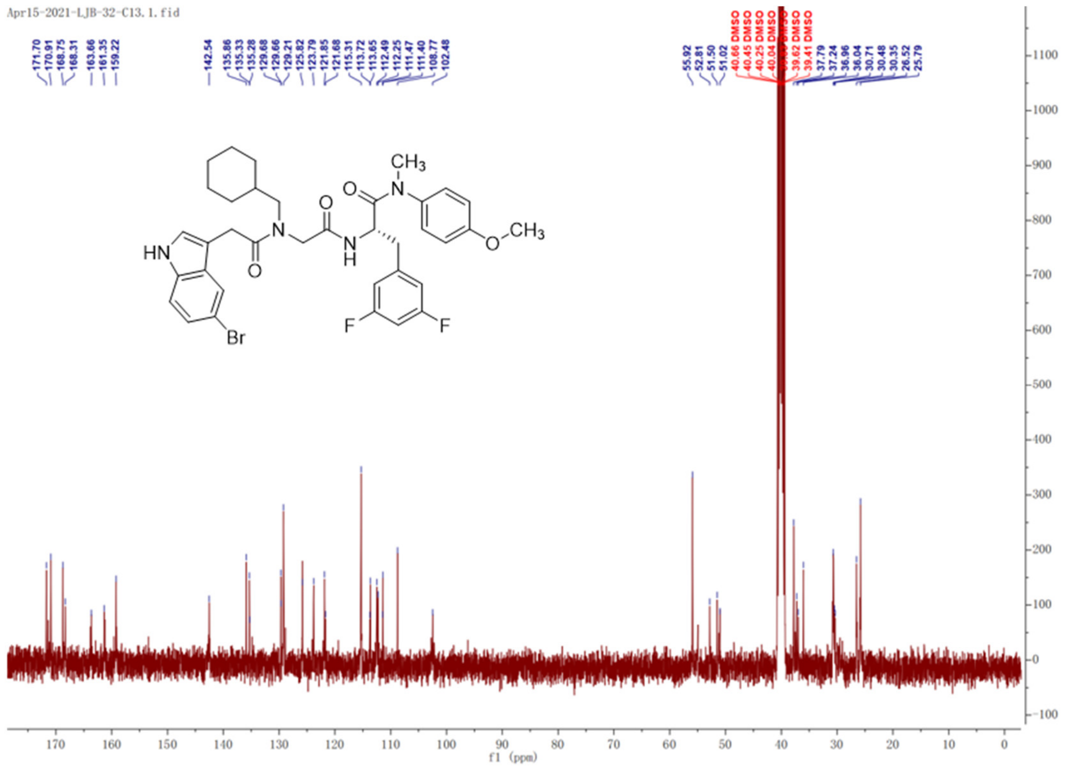

^{13}C-NMR of **I-10**

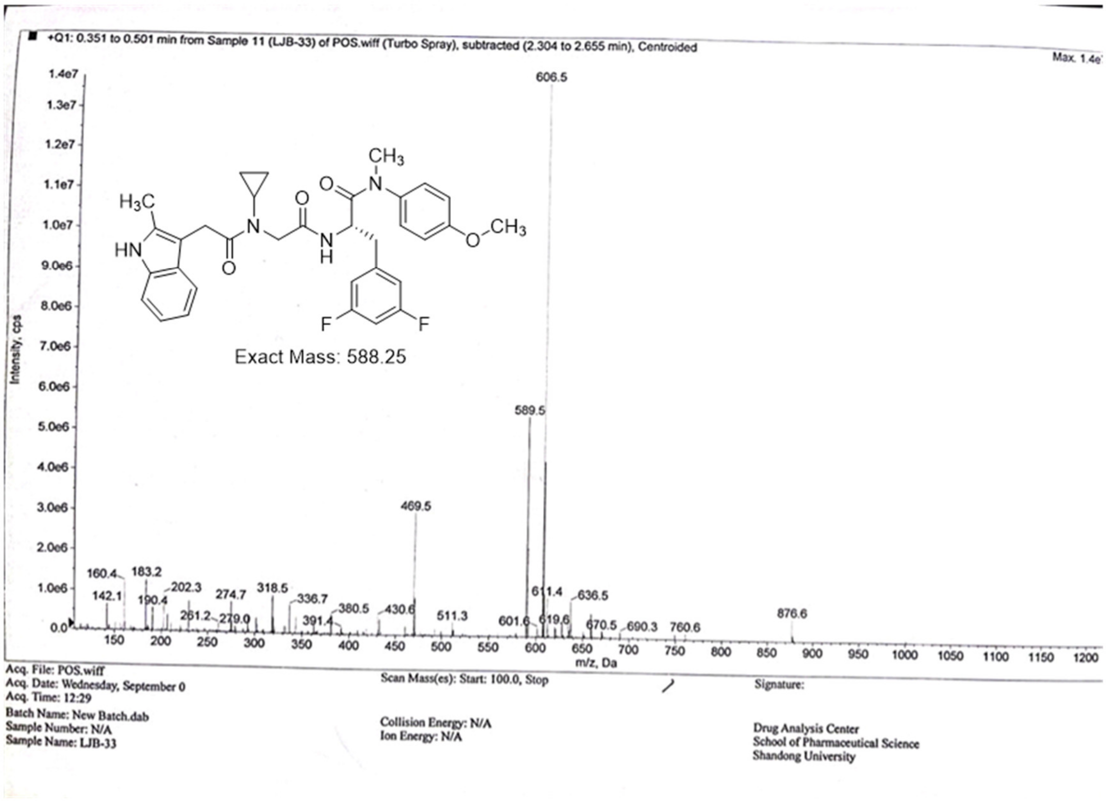

MS spectrum of **I-11**

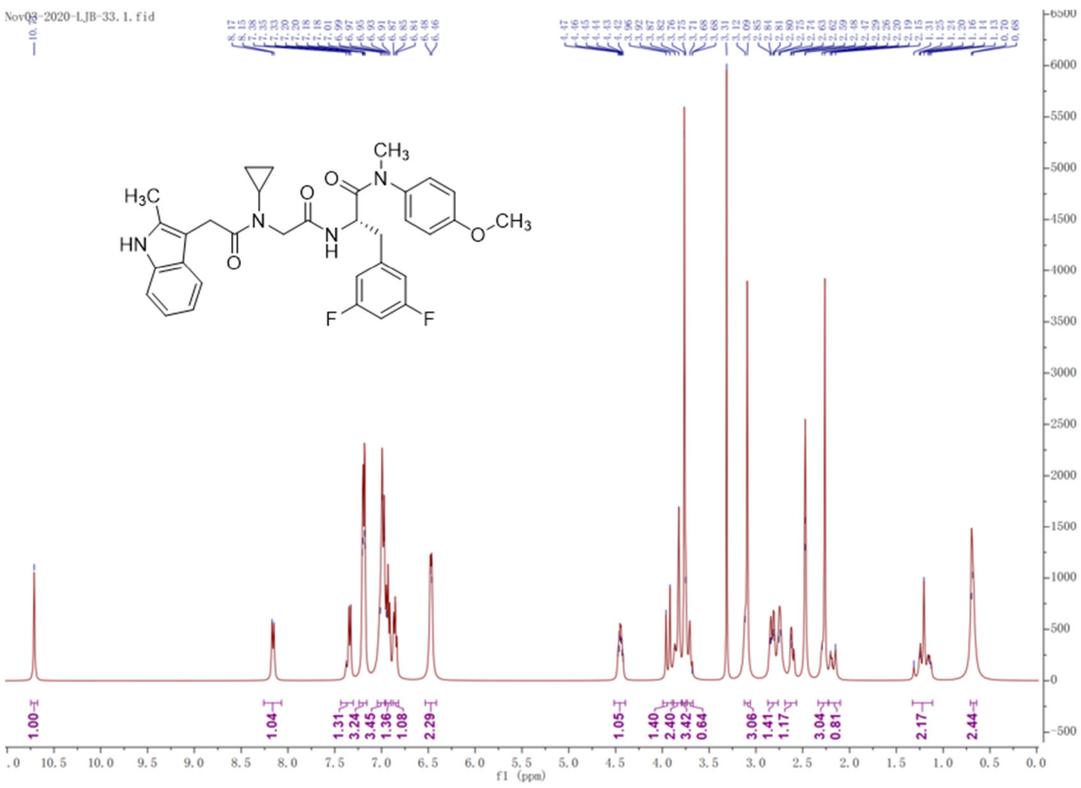

^{1}H-NMR of **I-11**

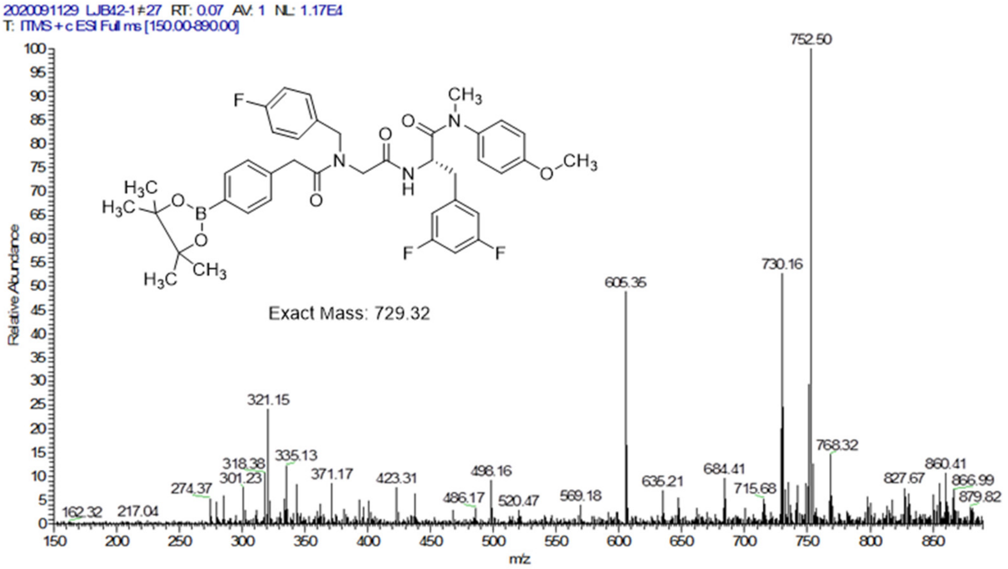

MS spectrum of **I-12**

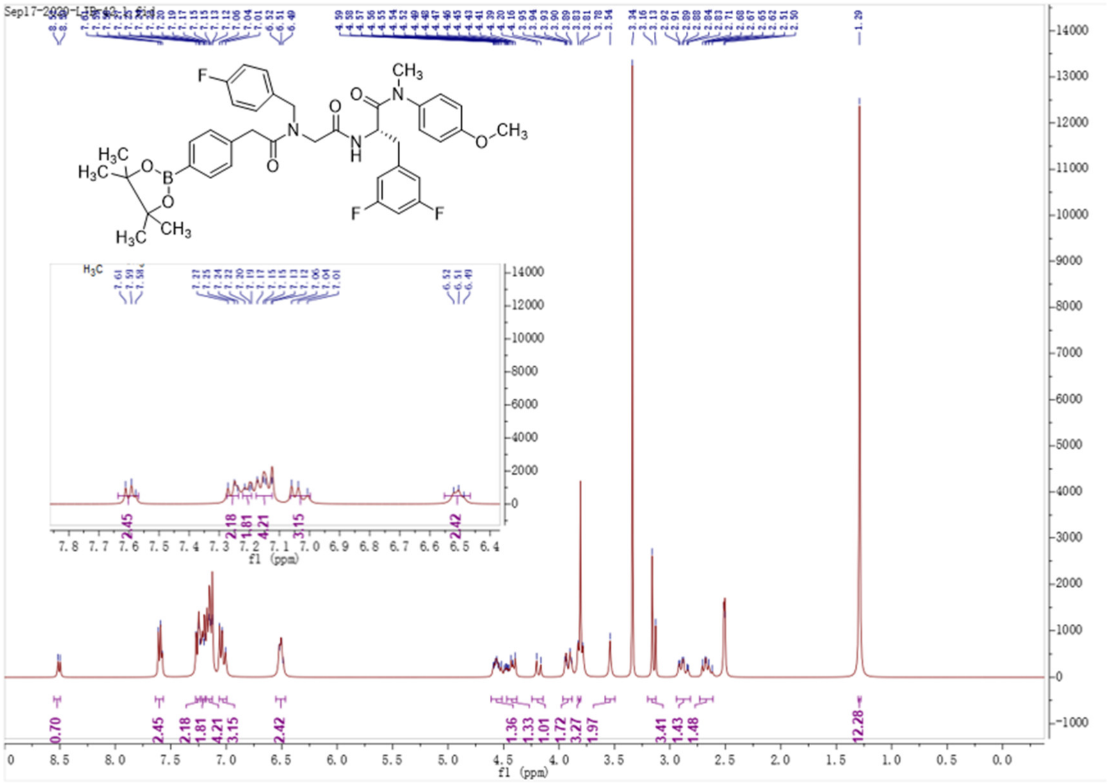

^{1}H-NMR of **I-12**

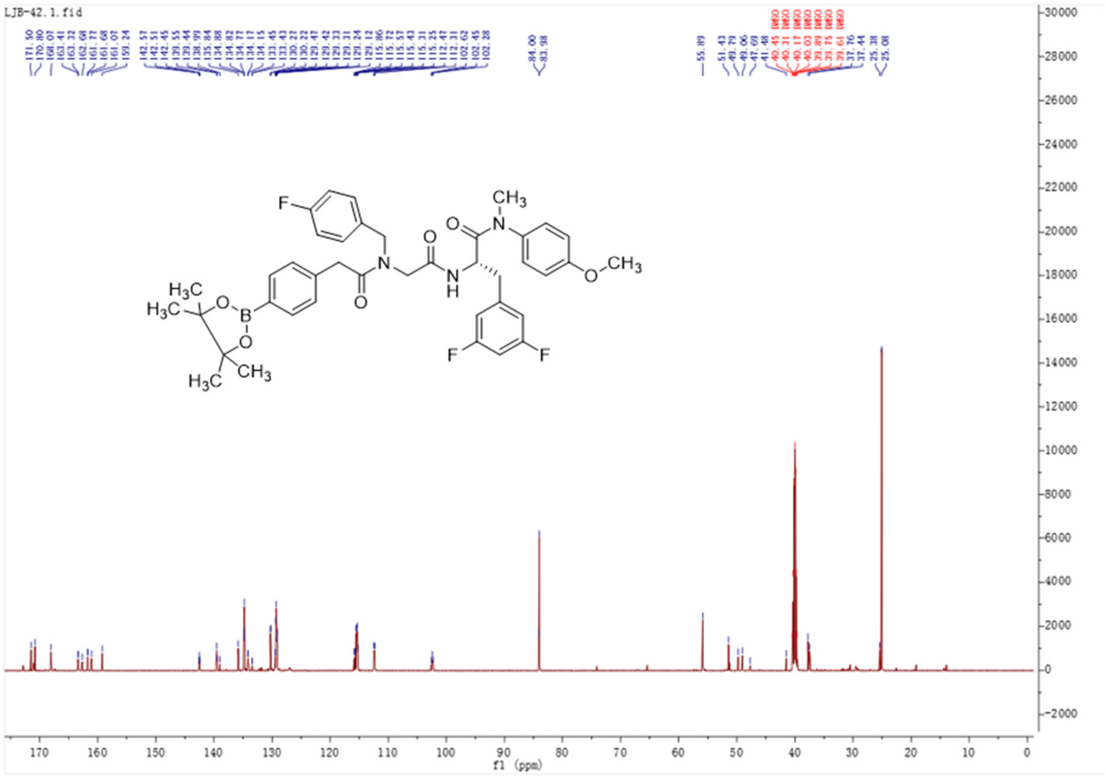

^{13}C-NMR of **I-12**

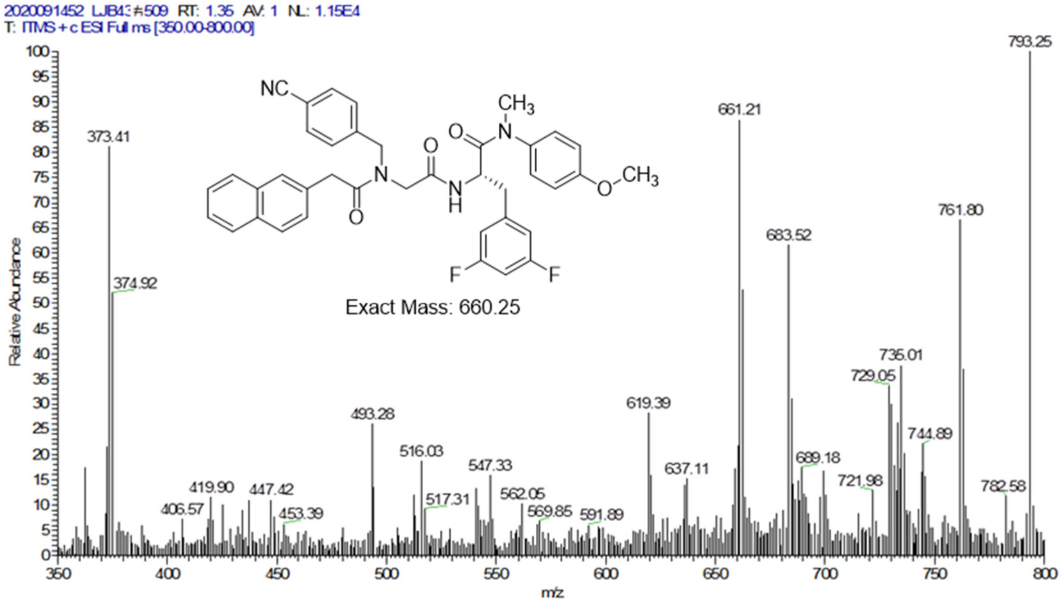

MS spectrum of **I-13**

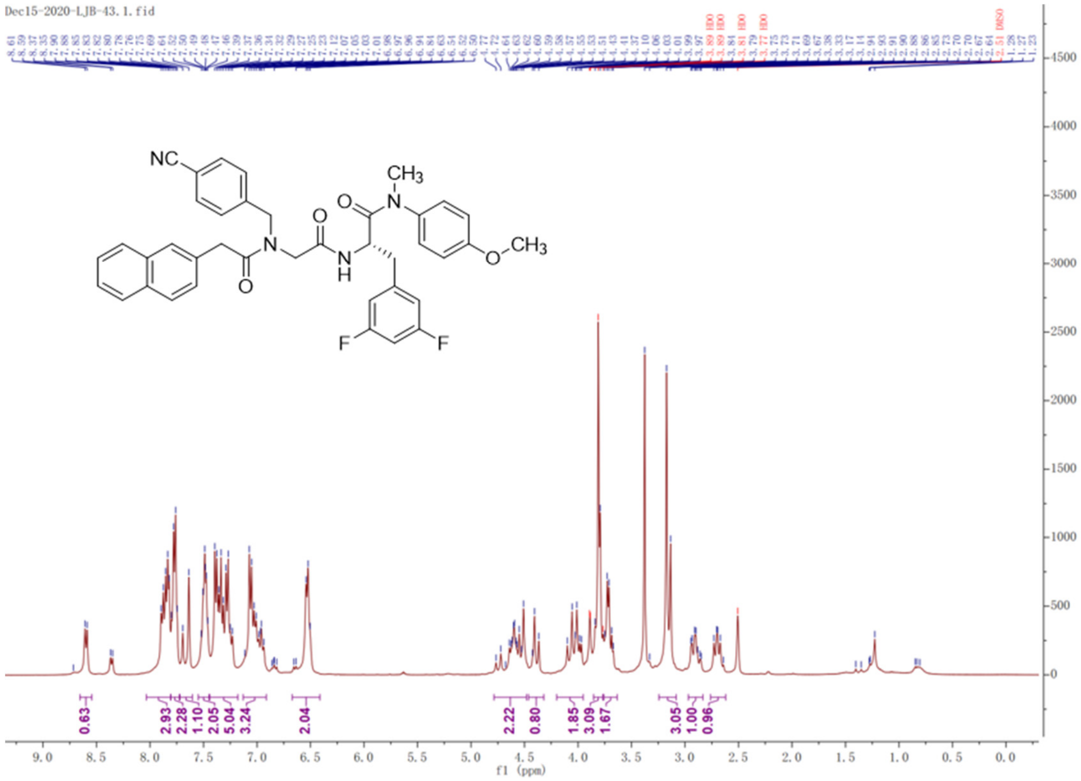

^{1}H-NMR of **I-13**

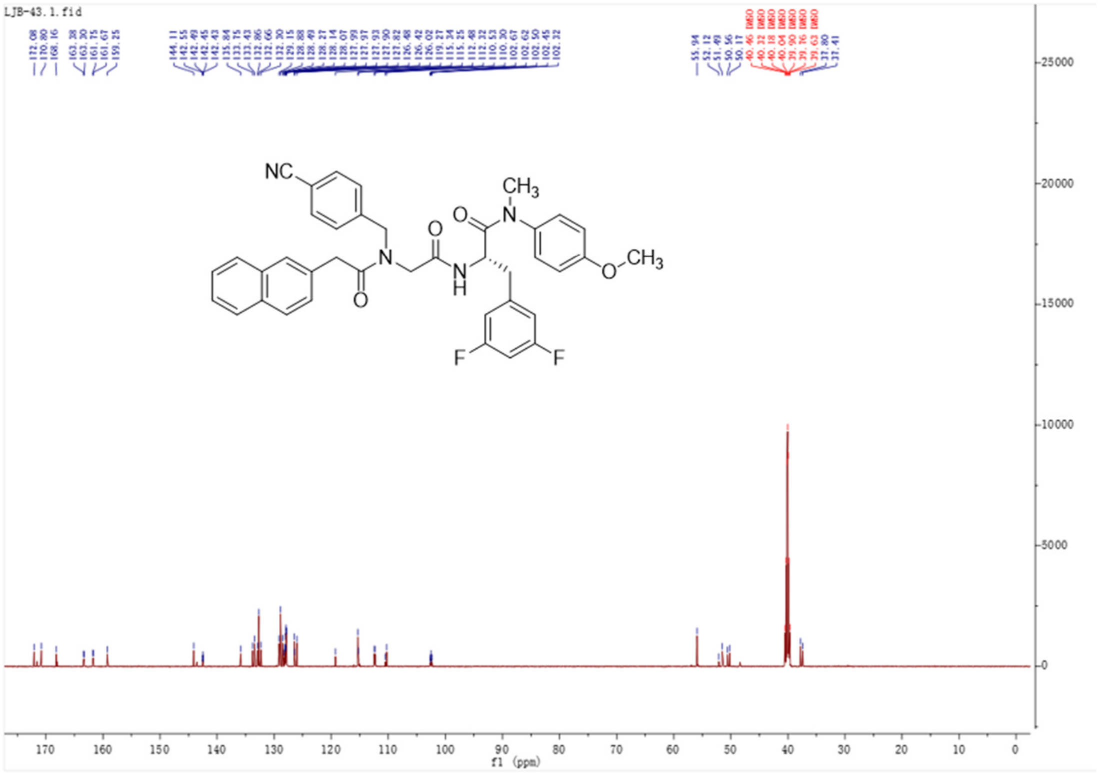

^{13}C-NMR of **I-13**

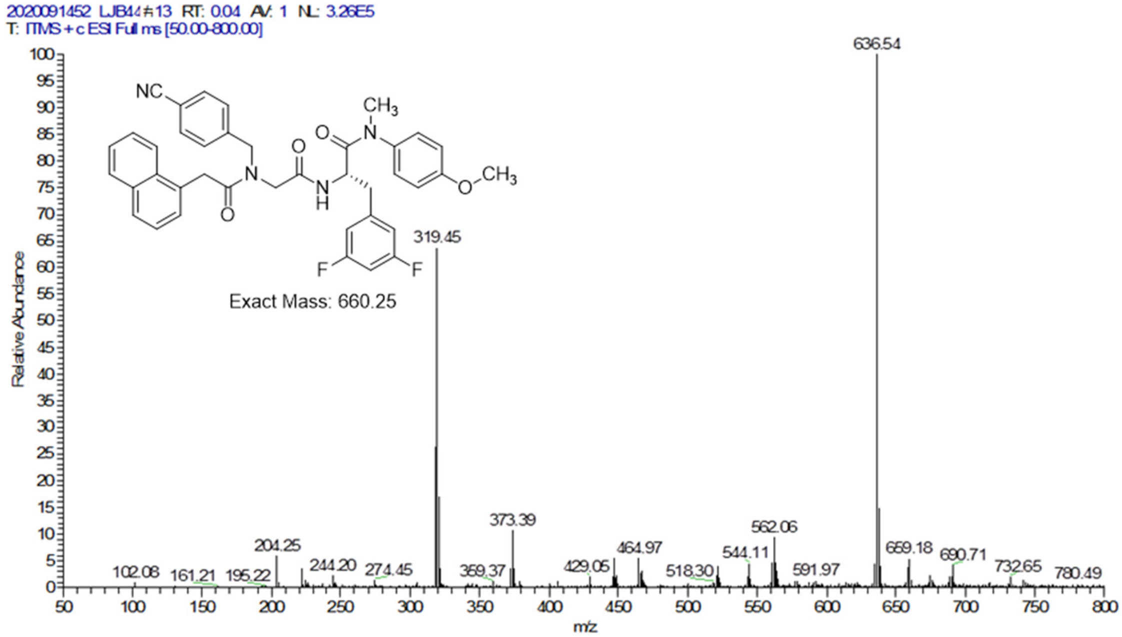

MS spectrum of **I-14**

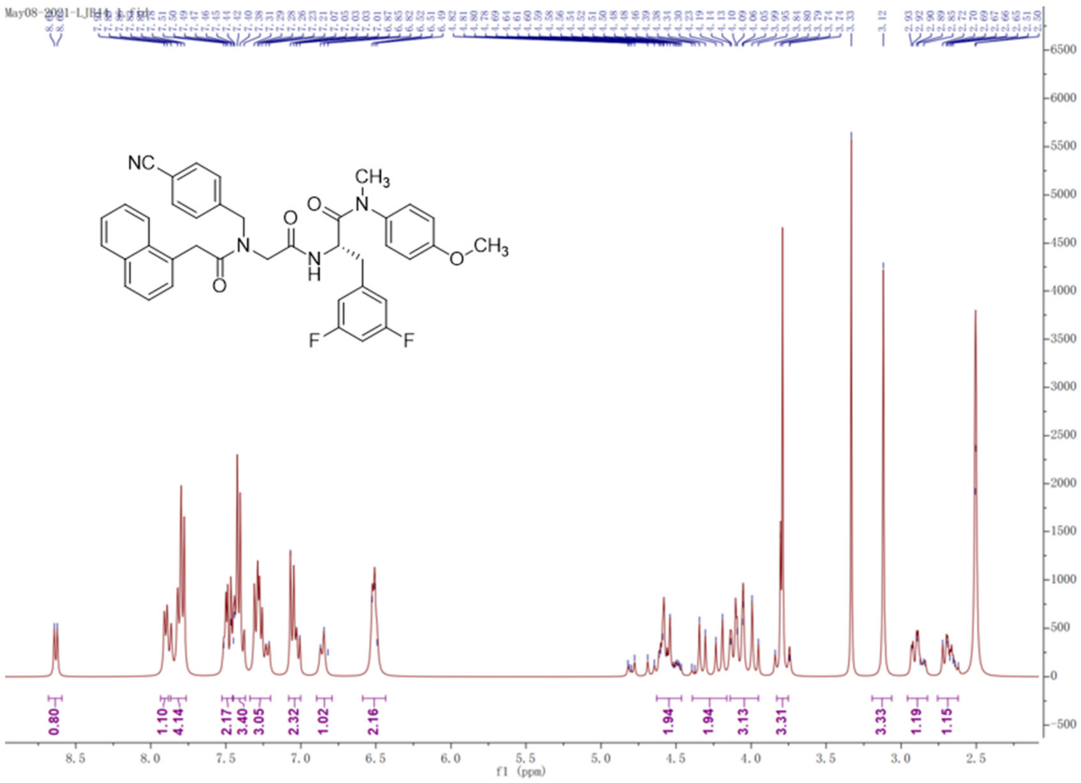

^{1}H-NMR of **I-14**

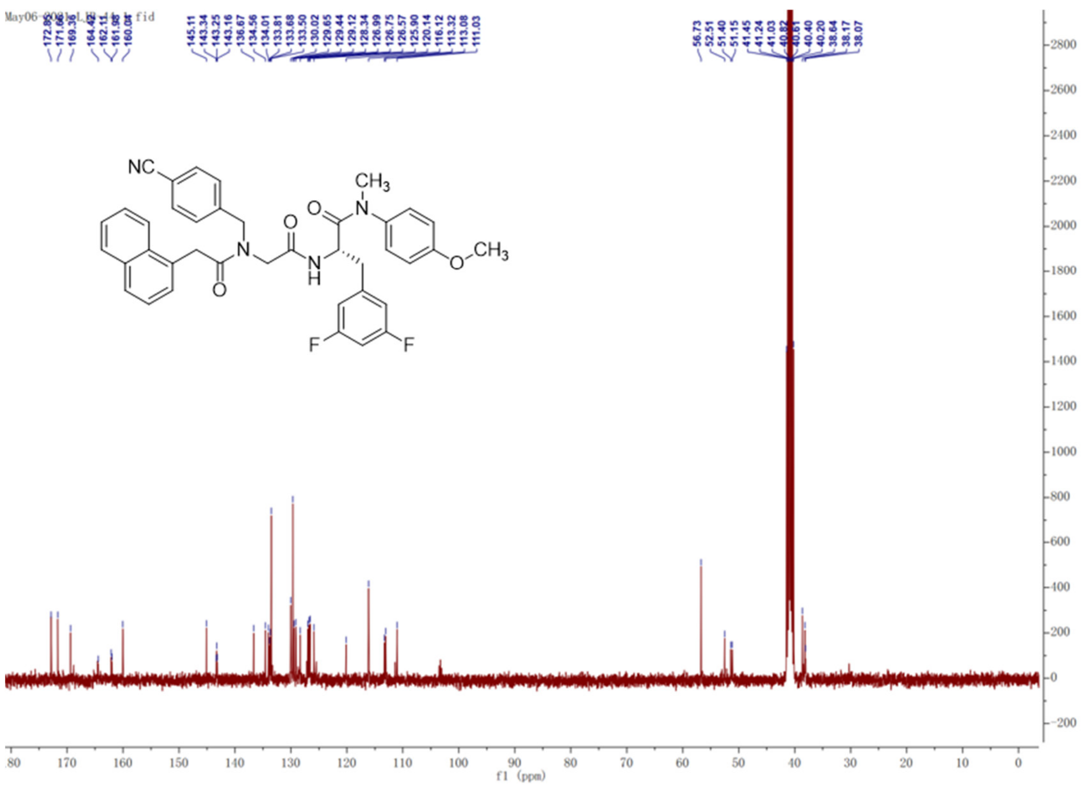

^{13}C-NMR of **I-14**

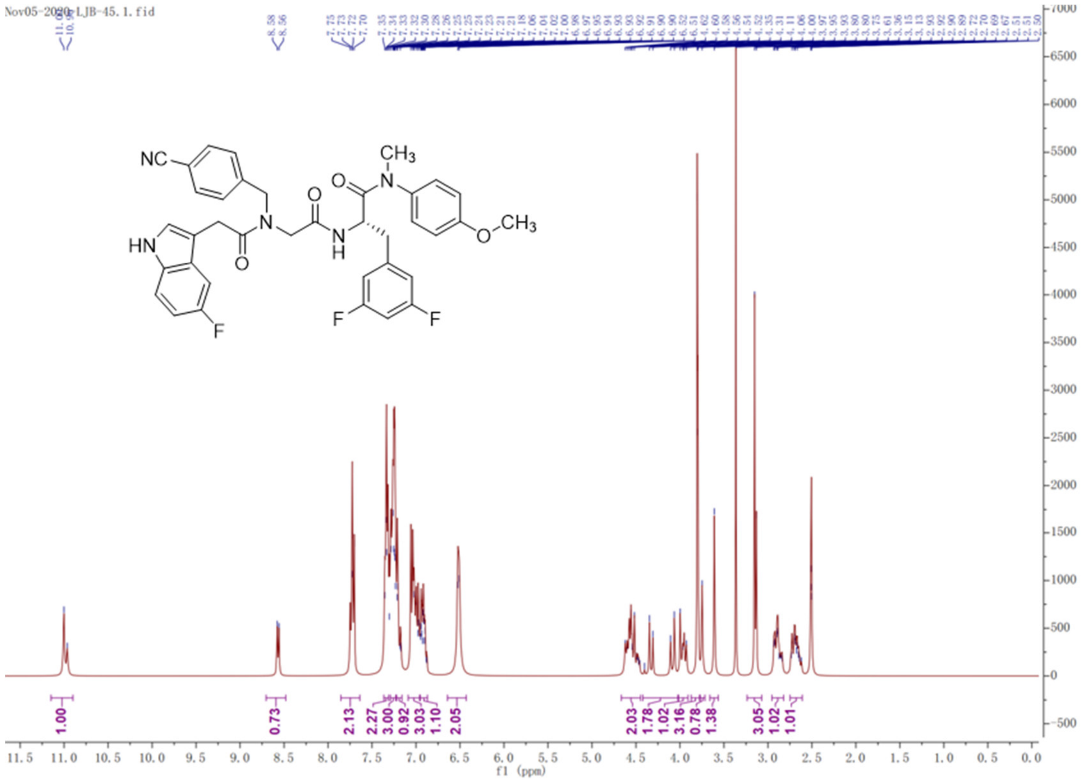

^{1}H-NMR of **I-15**

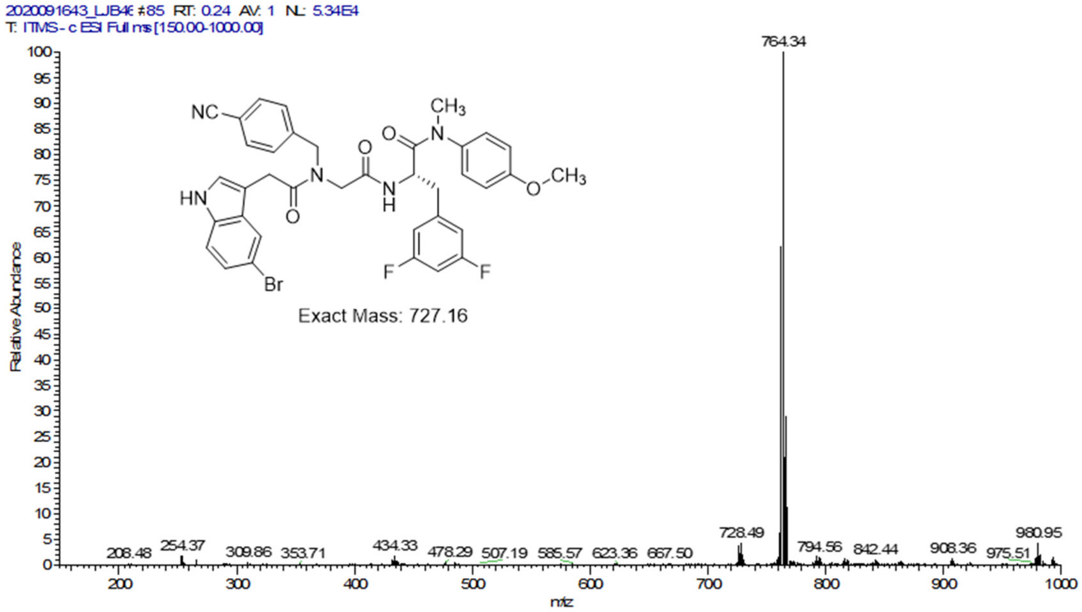

MS spectrum of **I-16**

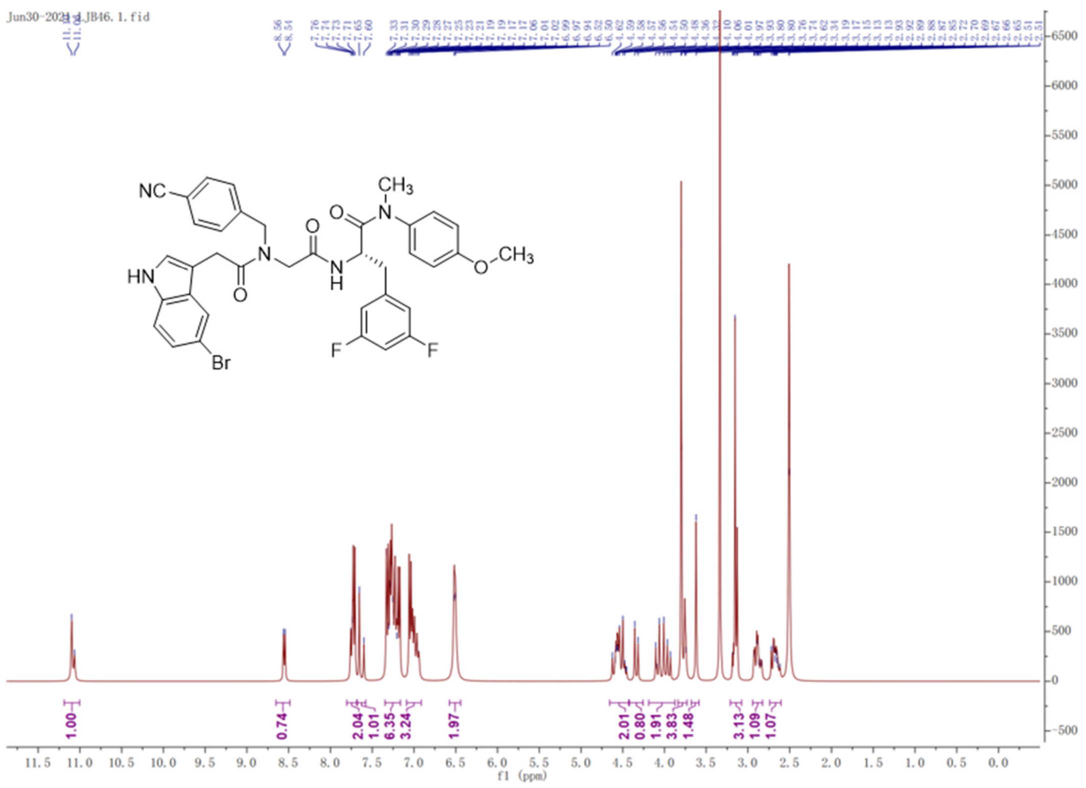

^{1}H-NMR of **I-16**

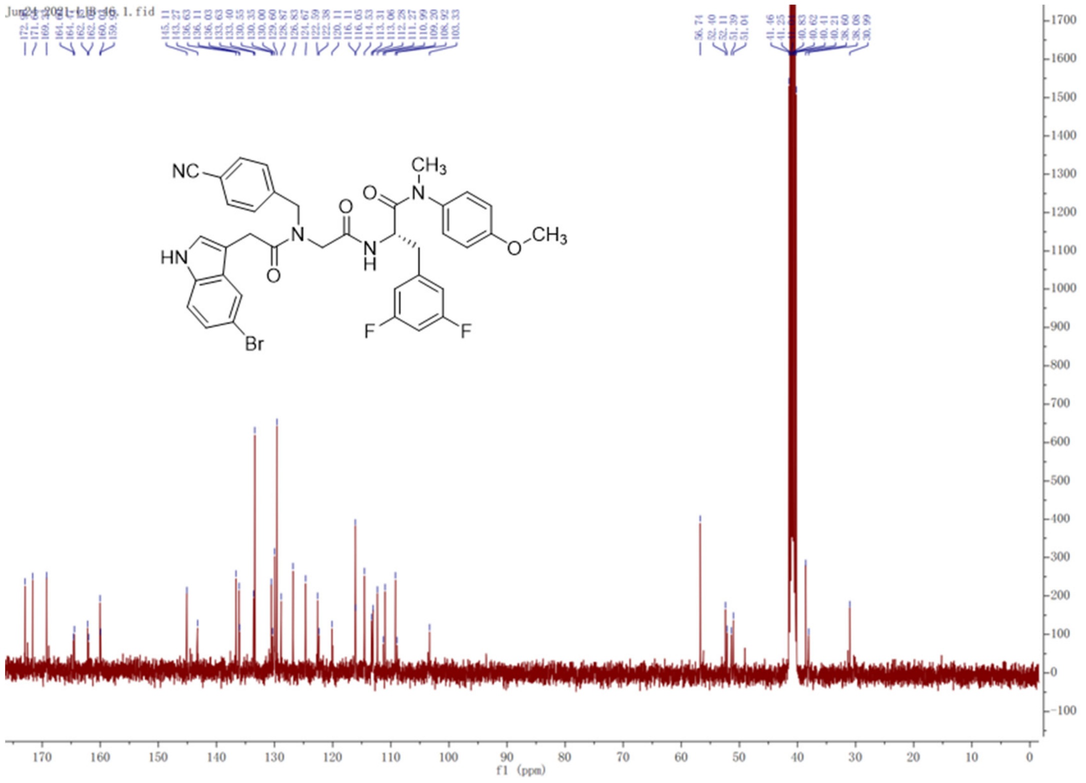

^{13}C-NMR of **I-16**

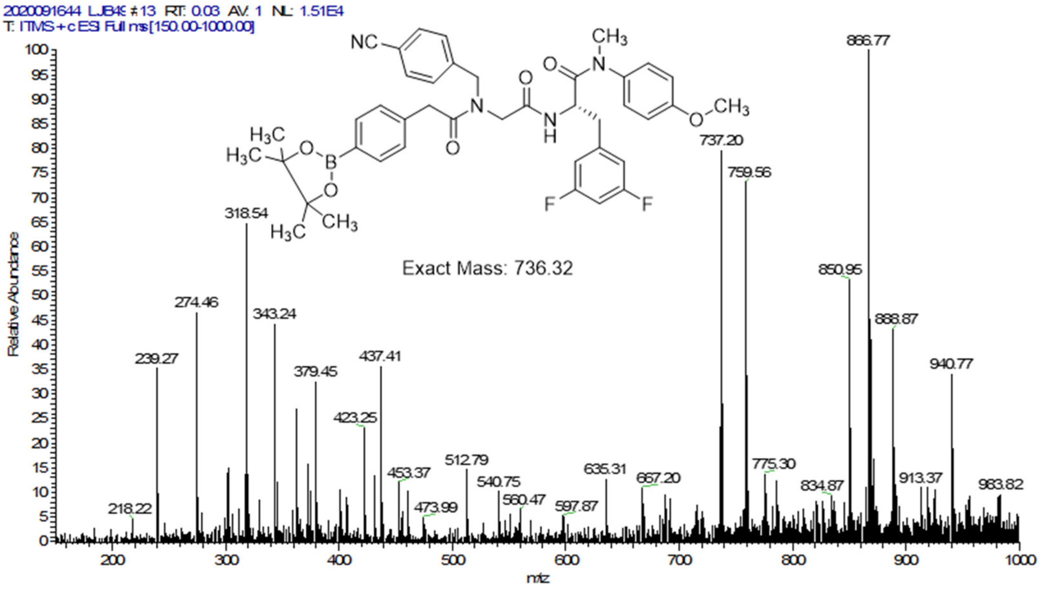

MS spectrum of **I-17**

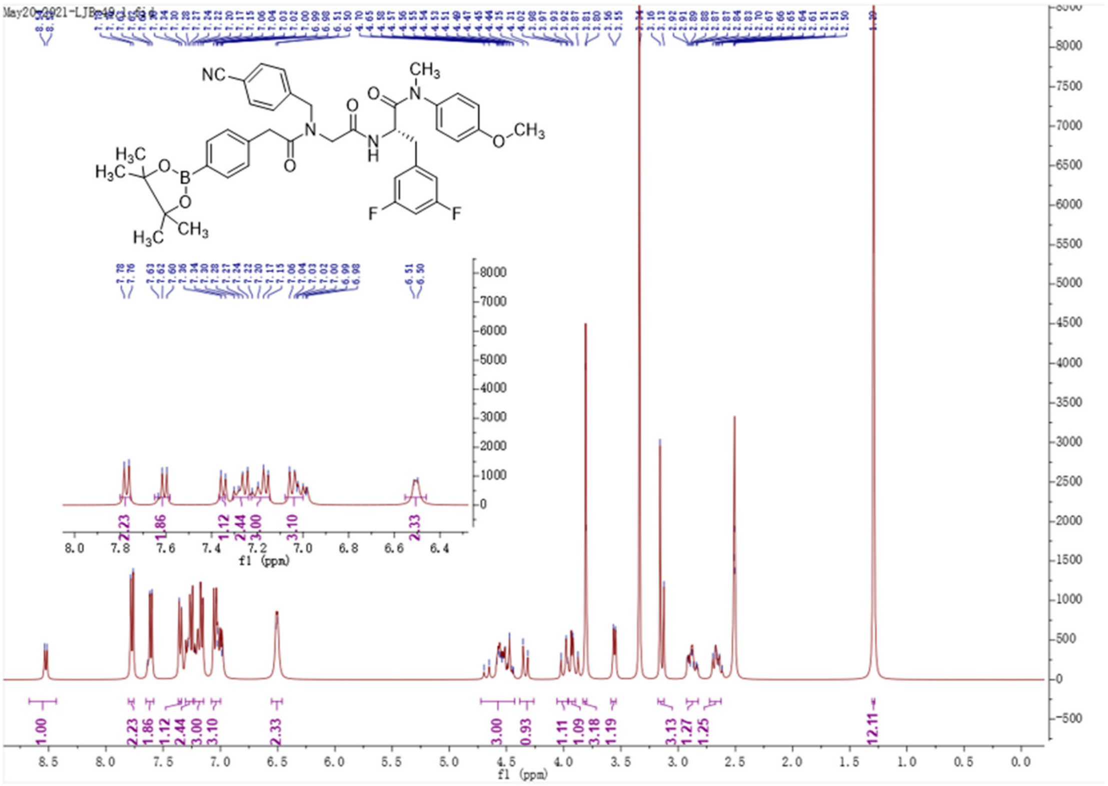

^{1}H-NMR of **I-17**

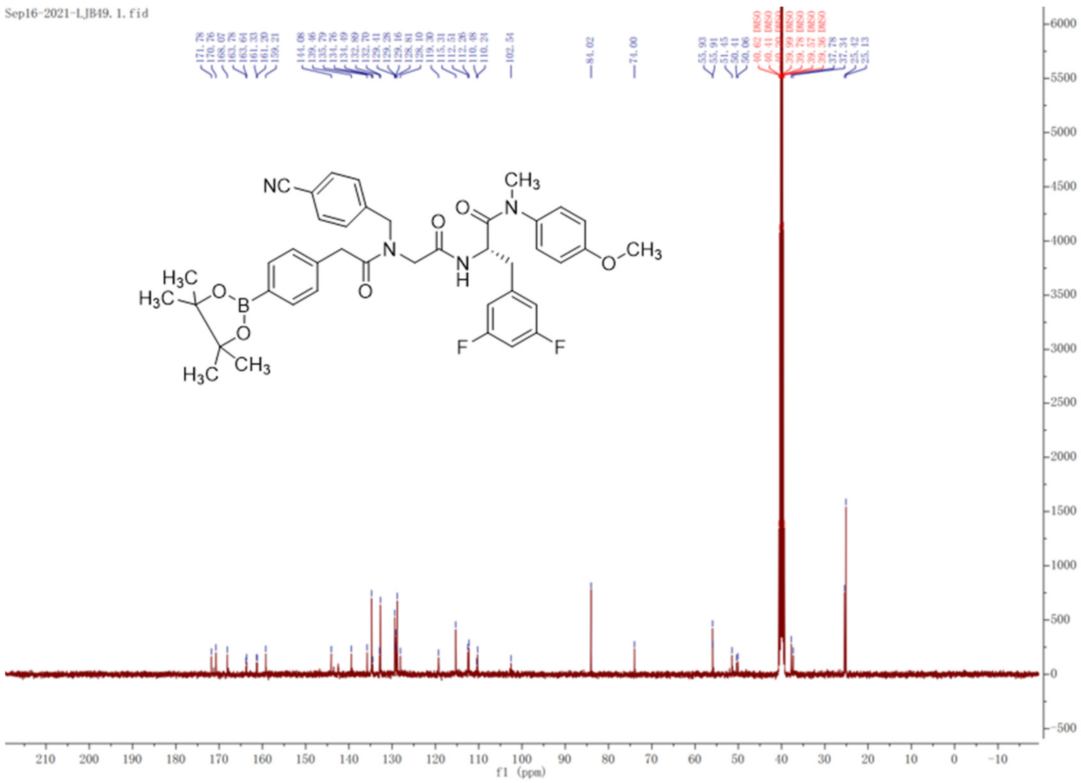

^{13}C-NMR of **I-17**

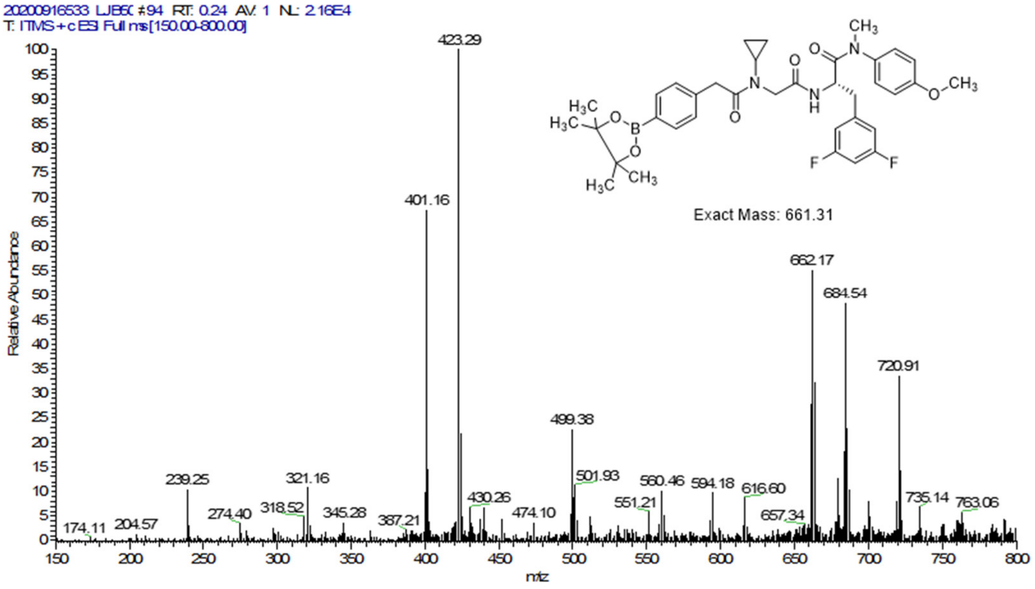

MS spectrum of **I-18**

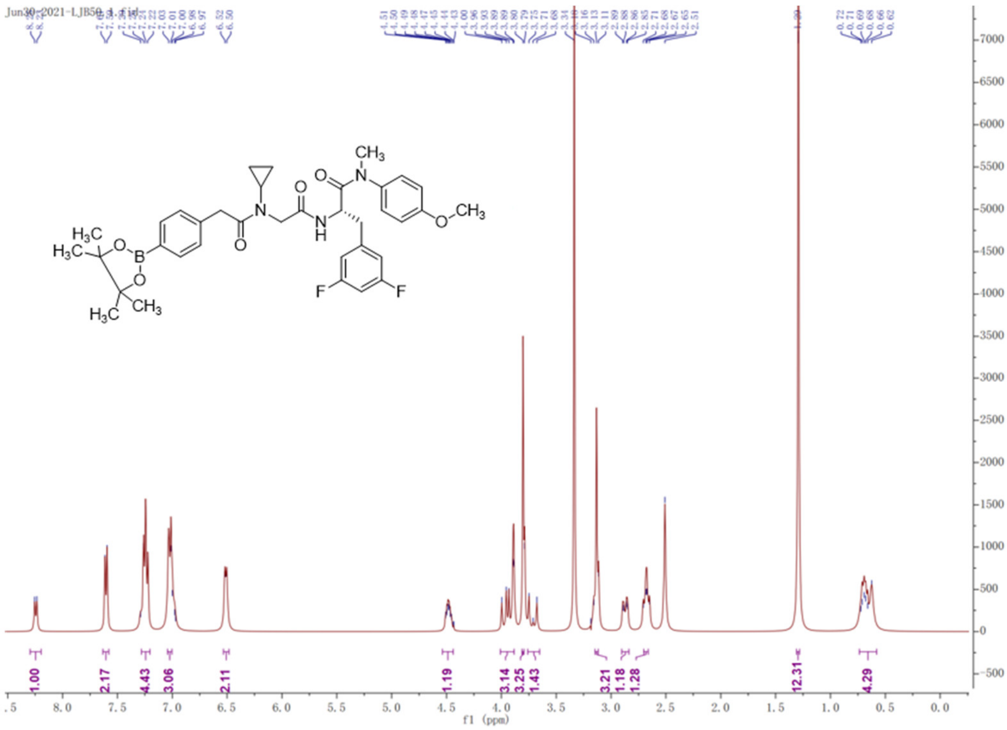

^{1}H-NMR of **I-18**

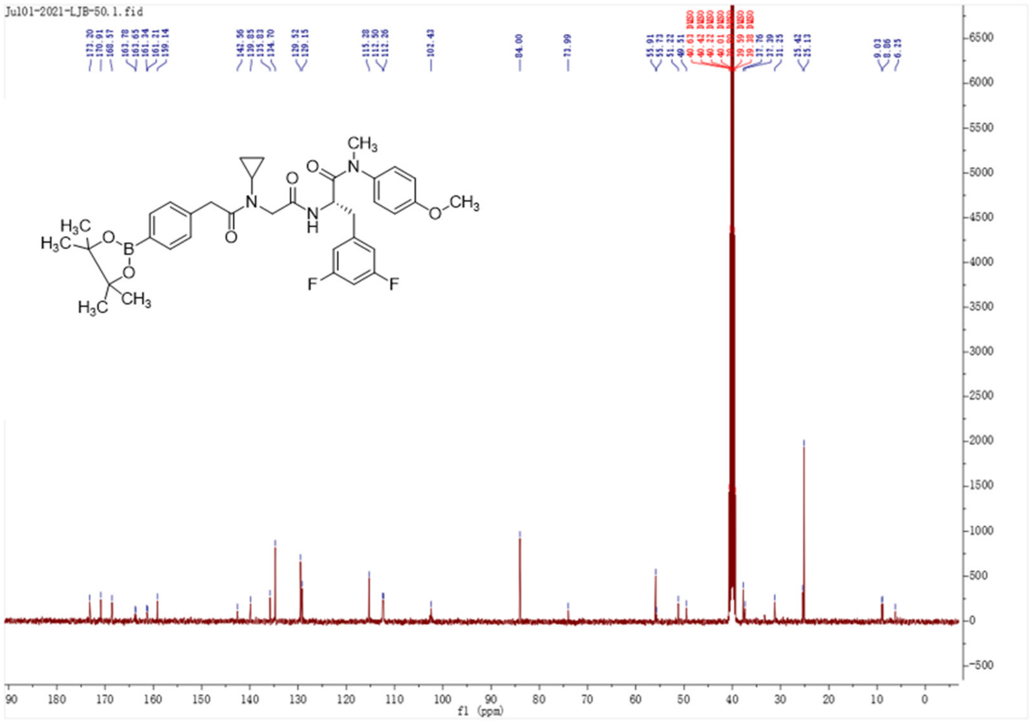

^{13}C-NMR of **I-18**

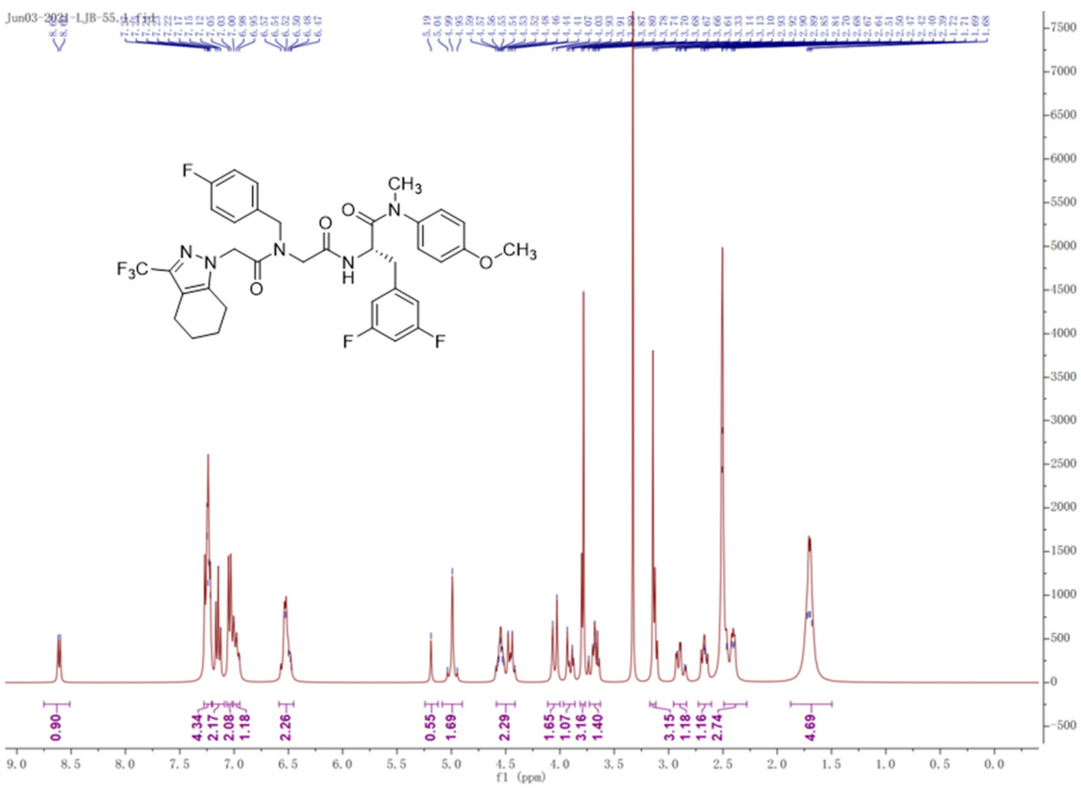

^{1}H-NMR of **I-19**

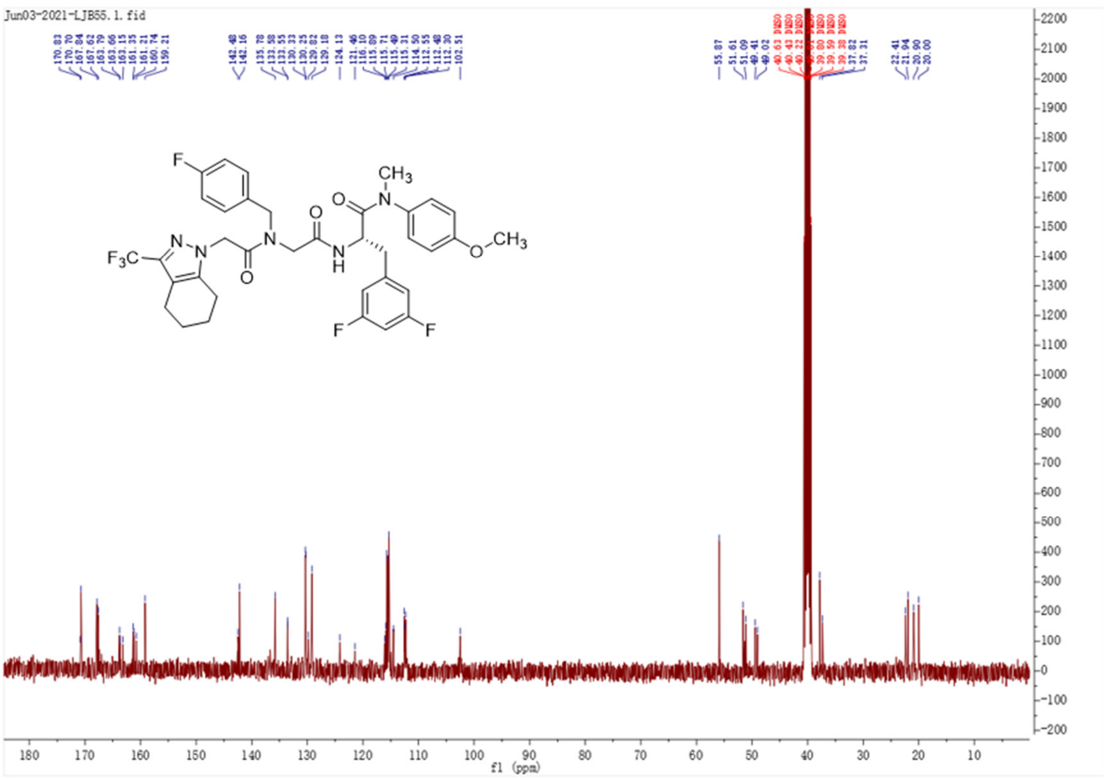

^{13}C-NMR of **I-19**

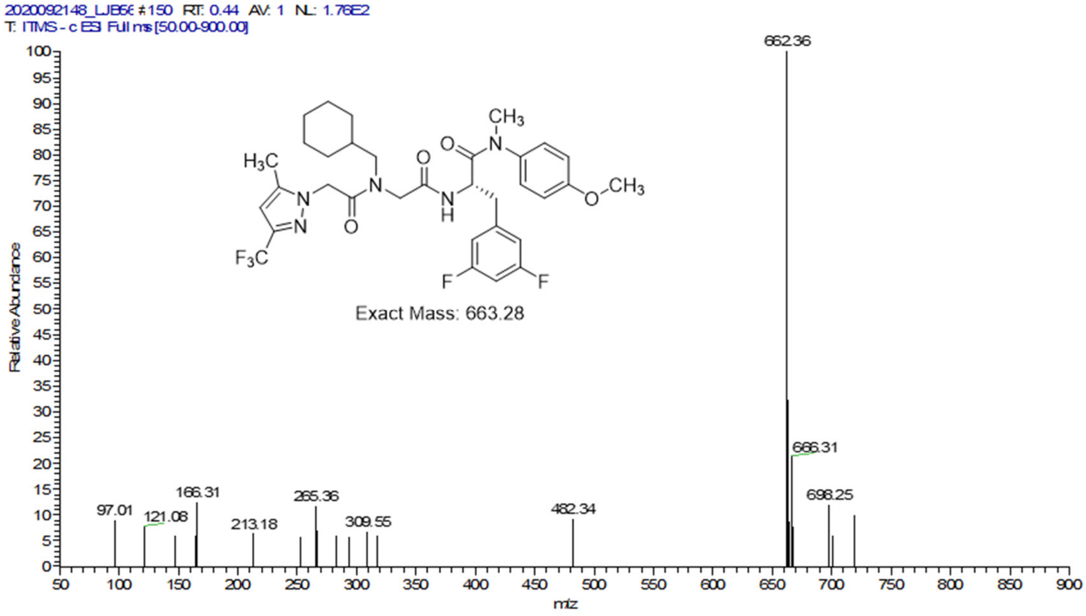

MS spectrum of **I-20**

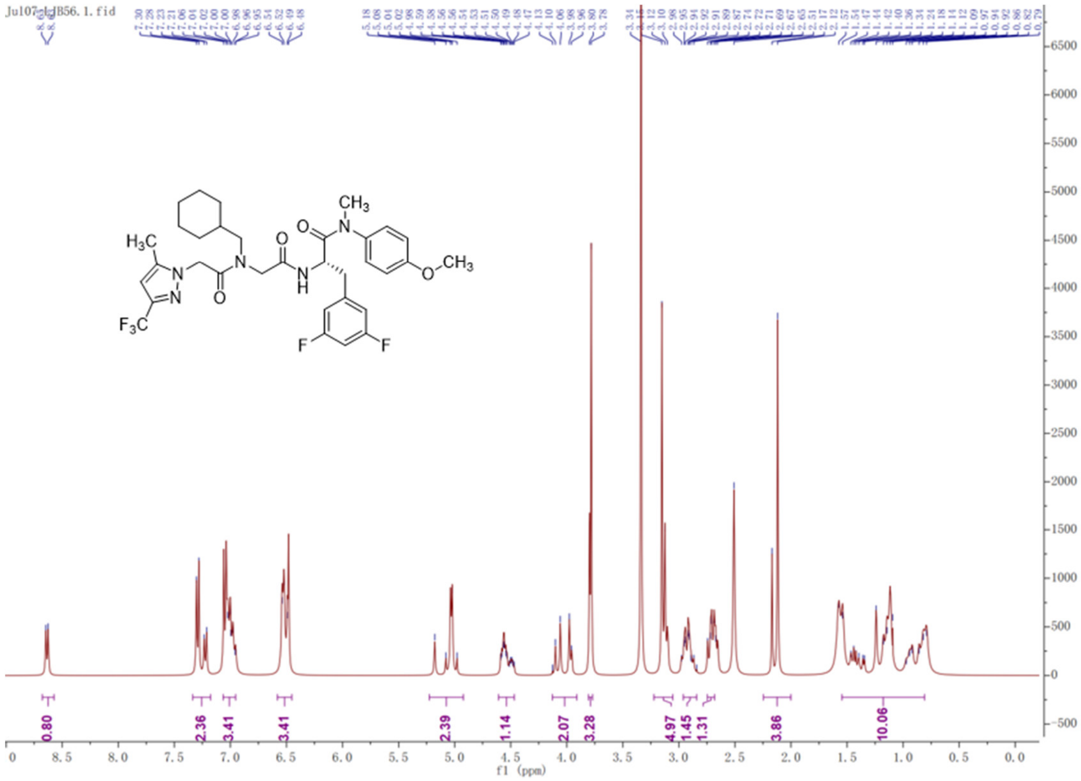

^{1}H-NMR of **I-20**

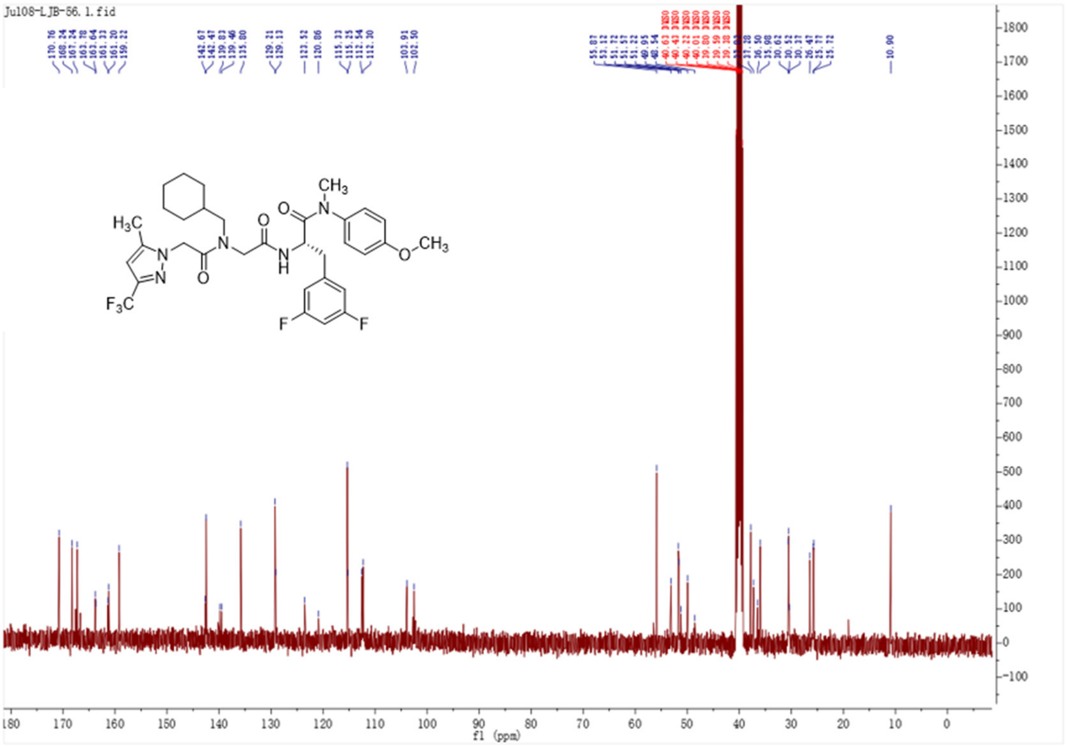

^{13}C-NMR of **I-20**

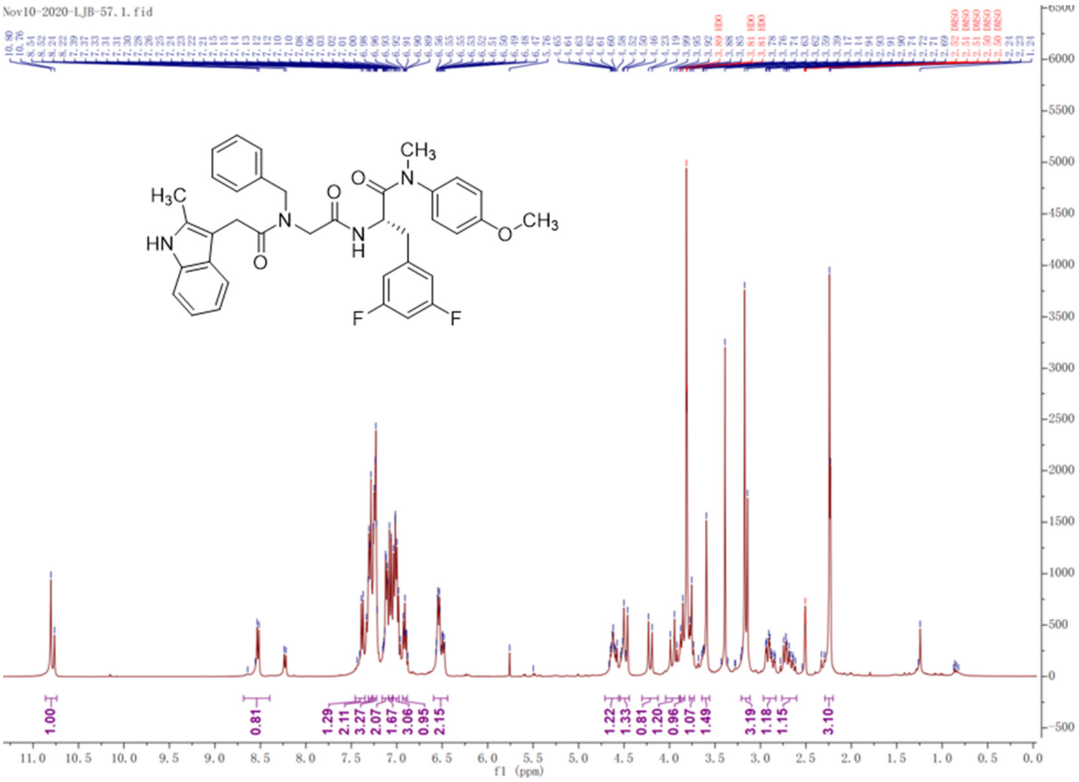

^{1}H-NMR of **I-21**

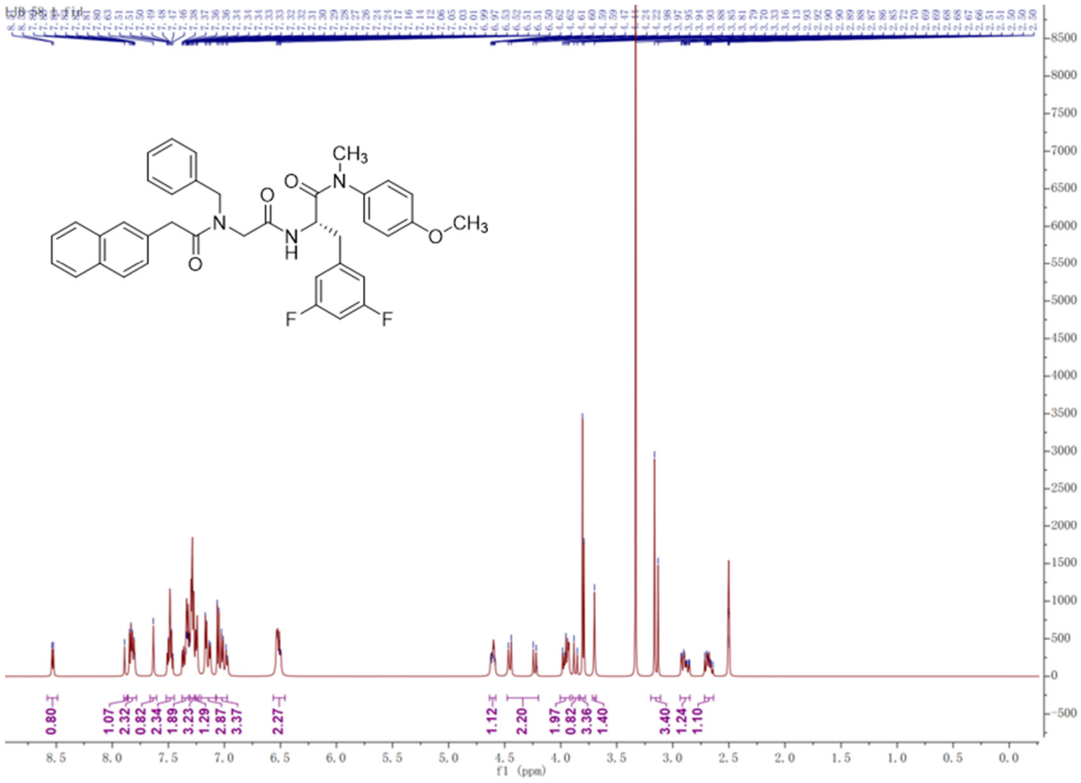

^{1}H-NMR of **I-22**

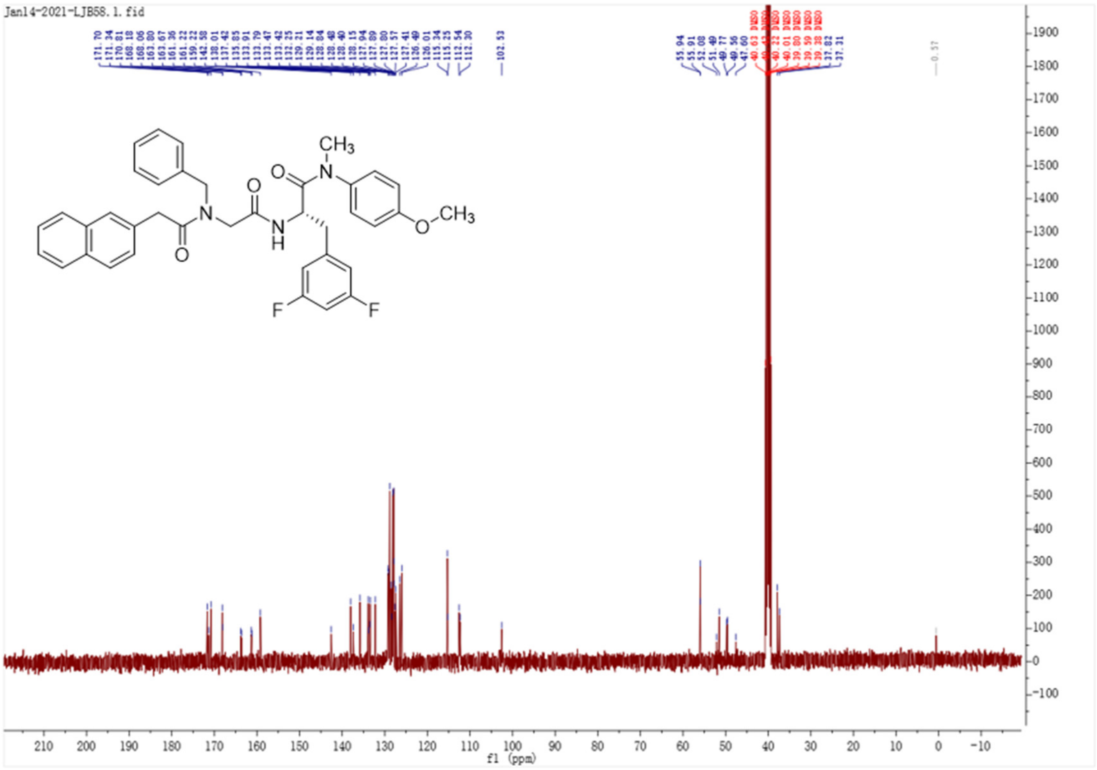

^{13}C-NMR of **I-22**

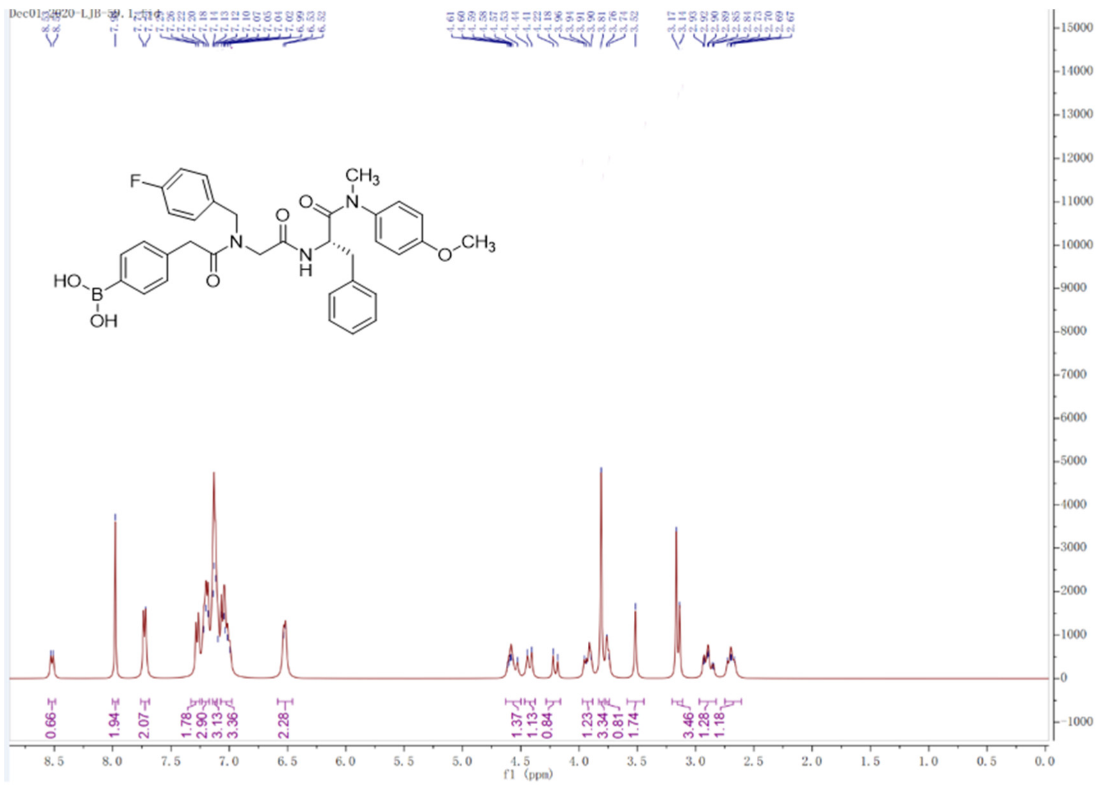

^{1}H-NMR of **I-23**

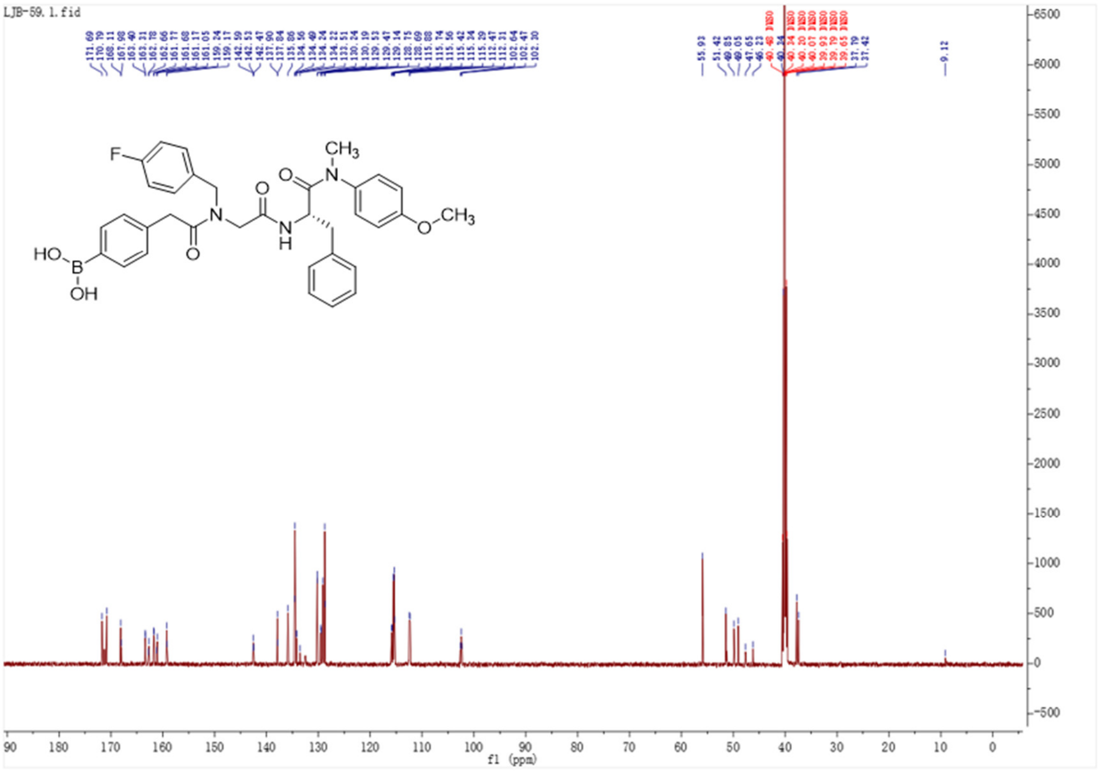

^{13}C-NMR of **I-23**
